# Combining palaeontological and neontological data shows a delayed diversification burst of carcharhiniform sharks likely mediated by environmental change

**DOI:** 10.1038/s41598-022-26010-7

**Published:** 2022-12-19

**Authors:** Baptiste Brée, Fabien L. Condamine, Guillaume Guinot

**Affiliations:** Institut des Sciences de l’Evolution de Montpellier, CNRS, IRD, EPHE, Université de Montpellier, 34095 Montpellier, France

**Keywords:** Evolution, Palaeontology, Phylogenetics, Ichthyology

## Abstract

Estimating deep-time species-level diversification processes remains challenging. Both the fossil record and molecular phylogenies allow the estimation of speciation and extinction rates, but each type of data may still provide an incomplete picture of diversification dynamics. Here, we combine species-level palaeontological (fossil occurrences) and neontological (molecular phylogenies) data to estimate deep-time diversity dynamics through process-based birth–death models for Carcharhiniformes, the most speciose shark order today. Despite their abundant fossil record dating back to the Middle Jurassic, only a small fraction of extant carcharhiniform species is recorded as fossils, which impedes relying only on the fossil record to study their recent diversification. Combining fossil and phylogenetic data, we recover a complex evolutionary history for carcharhiniforms, exemplified by several variations in diversification rates with an early low diversity period followed by a Cenozoic radiation. We further reveal a burst of diversification in the last 30 million years, which is partially recorded with fossil data only. We also find that reef expansion and temperature change can explain variations in speciation and extinction through time. These results pinpoint the primordial importance of these environmental variables in the evolution of marine clades. Our study also highlights the benefit of combining the fossil record with phylogenetic data to address macroevolutionary questions.

## Introduction

Estimating the dynamics of diversification rates and diversity variations represents a major challenge in evolutionary biology^1–3^. This is particularly paramount for testing a range of macroevolutionary questions^4–8^. Approaches have been developed to address this issue, each having their own specificities, limitations, and relevance considering the characteristics of the focal clade and hypotheses to be tested. Estimating diversification rates with phylogenies of extant taxa has proved to be powerful, especially as it allows for testing hypotheses related to trait evolution, biogeography and their links with deep-time diversity dynamics^9–11^. However, phylogeny-based approaches remain challenging and come with limitations on the taxon sampling, tree size, estimation of extinction, or on the limited identifiability of diversification processes^8,12–15^. Although they are useful for studying clades whose fossil record is poor and/or not preserving the biological traits to be studied, it has been shown that considering the fossil record is critical for addressing macroevolutionary questions^16–18^. In addition, many clades have no extant relatives (e.g. trilobites) or are represented today by very few taxa in comparison with their past diversity (e.g. coelacanths). In parallel, the use of the fossil record to infer diversification dynamics has historically been the subject of numerous works^19,20^. The incompleteness of the fossil record led to the development of various methods to correct observed times of speciation and extinction by trying to take into account sampling and preservation biases^2,3,21,22^. Bayesian approaches analysing fossil occurrences under a birth–death model have been developed to estimate preservation processes and times and rates of speciation and extinction^23^, which proved to be robust to estimate diversification patterns^24^. However, estimates based exclusively on the fossil record are not less challenging when we aim to connect extant and extinct diversities, simply because they are not sampled in the same way nor are they based on comparable data (molecular vs. morphological). Consequently, comprehensive estimates of diversification processes of extant clades require methods that allow combining fossil and phylogenetic data^25,26^.

Integrating palaeontological and neontological data has mostly relied on tree-based approaches^27^, including supertree^28^ or metatree^29^ methods. Alternatively, total-evidence dating^30^ or tip dating^31^ methods rely on the fossilised-birth–death model^32^ or the occurrence birth–death process^33^ to simultaneously infer a time-calibrated phylogeny including extant and extinct lineages in a single framework based on both morphological and molecular data (total-evidence dating) or morphology only (tip dating). While these approaches and their recent methodological developments offer a powerful phylogenetic framework to estimate past diversification events^34^, reconstructing such dated phylogenies still come with some methodological issues (e.g., effects of fossil sampling, missing data, settings of morphological clocks) that are not yet fully understood^35–38^.

A methodological gap remains when addressing the evolutionary history of extant clades for which phylogenetic frameworks including fossil taxa are lacking, as is the case for many clades, especially at lower taxonomic levels. The fossil record of some groups displays anatomical features allowing the building of a taxonomic scheme based on combinations of phenotypic character, but this type of data is hardly usable in broad phylogenetic analyses^39^. In addition, although the fossil record of many groups tends to be more complete towards the Recent—a characteristic known as the “pull of the Recent”^40^—some groups do not show this trend or even display an opposite pattern^41^. In the latter case, analyses exclusively based on the fossil record might not capture recent diversification events since extant and extinct diversities are disconnected, with most living taxa being not represented in the fossil record.

**Figure 1:**
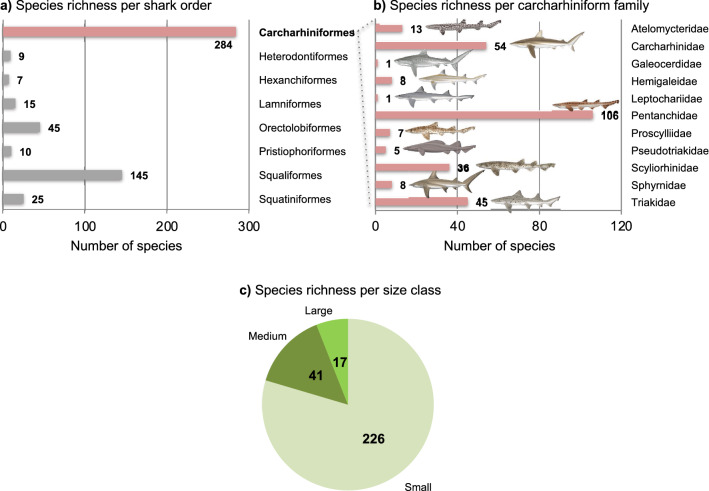
Biodiversity patterns in carcharhiniform sharks. (**a**) The order Carcharhiniformes stands out as the most species-rich shark order. (**b**) The species richness in carcharhiniform sharks is heterogeneously distributed across the 11 families ranging from one species (Leptochariidae) to 106 species (Pentanchidae). Carcharhiniformes also show great morphological diversity, for which the species richness is unevenly spread regarding tooth crown height (data on each species, see Supplementary Data S1). Shark images courtesy of Marc Dando (artist).

Carcharhiniformes are the most speciose shark order with ~ 290 living species included in nine families (Fig. 1)^42^. Molecular data are available for a large proportion of carcharhiniform species^43^, but comprehensive time-calibrated species-level phylogenies are scarce for the entire order^43,44^. This clade has a long evolutionary history, originating in the Middle Jurassic (ca. 170 Ma), and a rich fossil record across most of this timespan. As in all elasmobranch species, tooth replacement is continuous (polyphiodonty), which results in an abundant production of dental remains that are composed of hard tissues with a high preservation potential. Consequently, the carcharhiniform fossil record is mainly represented by isolated teeth whose anatomical features provide valuable information for taxonomic purposes. However, characters provided by tooth morphology are difficult to use in morphological phylogenetic or total-evidence dating analyses for most elasmobranch orders, although some attempts on few elasmobranch clades with derived tooth morphology proved conclusive^45,46^. This challenge is mainly due to the tremendous diversity of dental forms related to the large range of ecologies and feeding strategies within and between orders, making the definition of common morphological characters and identification of homologous structures difficult^47^. Resolving species-level morphology-based phylogenetic relationships of extant elasmobranchs is an ongoing challenge due to the large diversity of the clade, frequent description of new species, and lack of internal anatomical data for most species^41,48^. Consequently, although some attempts have been made to include fossil elasmobranch taxa within a phylogenetic framework at high taxonomic levels to explore diversification patterns^49,50^, Bayesian total-evidence dating remains difficult to perform at the species level. Furthermore, despite the abundant elasmobranch fossil record, only a very small fraction of extant carcharhiniform genera and species are recorded as fossils^41^. This mirrors incomplete knowledge on species-level tooth morphology of extant taxa that complicates species delimitations in the recent fossil record, coupled with weaker sampling of post-Miocene (< 5 Ma) geological formations, which departs from the ‘pull of the Recent’^51^. Consequently, although the carcharhiniform fossil record allowed species-level estimates of diversification events, the probable post-Paleocene diversification of the clade is partly hidden when only considering fossil data^52^.

**Figure 2:**
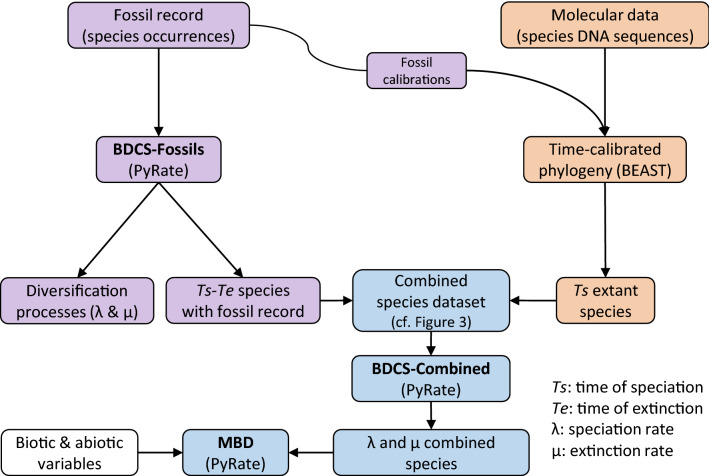
Schematic representation of the workflow of the study. We compiled the fossil record (purple colour) and molecular data (orange colour) at the species level for Carcharhiniformes that were analysed separately and jointly (blue colour). The fossil occurrence data are first analysed under a birth–death with constrained shifts (BDCS-Fossils) model, using PyRate, while jointly modelling the preservation process to estimate speciation (λ), extinction (μ), and preservation (*q*) rates. This also estimates the times of speciation (*Ts*) and times of extinction (*Te*) of all species with a fossil record. The DNA sequences were analysed with Bayesian phylogenetic inferences, using BEAST, while simultaneously estimating the tree topology and divergence times using fossil calibrations retrieved from the fossil compilation. The divergence times provide *Ts* for extant species that have no fossil record (see Fig. 3 for details on this step). We then combined the *Ts* and *Te* estimated with PyRate and the *Ts* and *Te* inferred with molecular dating in BEAST to generate the most comprehensive species-level dataset incorporating *Ts* and *Te* as well as the age uncertainties. This dataset is used for the subsequent analyses under the BDCS model (BDCS-Combined) to compare diversification dynamics with the fossil-only estimates, and the multivariate birth–death (MBD) model to assess the role of global environmental change, as well as clade competition on speciation and extinction rates.

Here we aim to provide a comprehensive estimation of carcharhiniform diversification history by combining palaeontological and neontological data (Fig. 2; see “Methods” below). First, we compiled available molecular data (14 gene fragments) to reconstruct a Bayesian species-level time-calibrated phylogeny of Carcharhiniformes, from which we extracted divergence times of extant species taking into account age uncertainties. Second, we compiled species-level fossil occurrences (> 1300) of the whole clade, spanning the entire existence of the order, to estimate fossil occurrence-based diversification rates with Bayesian process-based birth–death models incorporating the temporal variation of the preservation processes and uncertainties associated with the age of fossil occurrences as implemented in PyRate^24^. Third, we combined the phylogeny-based and fossil occurrence-based estimates of extant and extinct species’ lifespans into a single Bayesian framework implemented in PyRate to provide inclusive estimates of the carcharhiniform diversification processes through time. Fourth, we assessed whether the speciation and extinction rates of carcharhiniforms responded to major crises such as the Cretaceous-Paleogene (K-Pg) event and Eocene–Oligocene transition (EOT), to environmental changes in Earth history, and to intra-clade biotic interactions^53^. We also performed a comparison of the macroevolutionary history of carcharhiniforms between an inference drawn from the fossil record only and an inference made with the combined dataset. We argue that combining extinct and extant taxa based on different types of datasets (fossil occurrences and molecular phylogenies) offers an opportunity to provide more reliable estimates of the evolutionary history of clades experiencing recent radiations that correspond to periods of undersampled fossil record.

## Methods

### Taxon sampling, molecular data, and phylogeny

#### Extant species

The species-level systematics and phylogeny of the order Carcharhiniformes is currently unstable for most clades^54,55^. This situation may be explained by the tremendous diversity of the clade, lack of biological data and description of the internal anatomy for numerous species, and high number of recently described species (more than 25 species described over the past decade), as in most elasmobranch clades^41^. In addition to the weak morphological support, some species are based on non-adult specimens^56^ and/or a single specimen^57,58^, and molecular data are not systematically produced upon descriptions of new species, or remain inaccessible to other researchers. Consequently, we reviewed the validity of all extant carcharhiniform species based on literature and mainly followed Weigmann^59^. We established a list of 284 species that we consider as valid and provided a list of species considered as non-valid or requiring revision (Supplementary Data S1).

The latest comprehensive phylogeny of sharks^43^ generated a molecular supermatrix composed of two non-protein-coding mitochondrial loci (12S and 16S ribosomal DNA), 11 protein-coding mitochondrial loci (CO1, CO2, CO3, Cyt b, ND1, ND2, ND3, ND4, ND4L, ND5 and ND6) and two nuclear protein-coding loci (RAG1 and SCFD2). We collected all molecular data of Carcharhiniformes available on GenBank and BOLD Systems (last accessed on 18 June 2020) and retrieved DNA sequences from the same 13 mitochondrial genes and one nuclear locus (RAG1). We did not gather the data for the nuclear gene SCFD2 because the species sampling was too low (< 5%) for Carcharhiniformes. The complete mitochondrial genome was recovered for 44 species from which we extracted the 13 mitochondrial genes. Data completion was variable among loci from 18% of the species for RAG1 to 87% of the species for COI. All families of Carcharhiniformes were included, as well as 50 genera and 195 species, representing 96.2% of the generic diversity and 68.7% of the species diversity of the order, respectively. DNA sequences for some carcharhiniform species could not be included in our analyses, although they were used in previously published works^56,60,61^. These sequences were not available through public repositories and were not made available to us despite formal requests to the authors. The resulting dataset was substantially improved compared with the last shark supermatrix published^43^, which included 55.63% of the total extant carcharhiniform species diversity. We selected all 15 extant species of the order Lamniformes, a clade constantly found in sister position to the order Carcharhiniformes^43,55^, as outgroup. The accession numbers for all the DNA sequences of the dataset are available in the Supplementary Data S2.

#### Phylogeny and dating

Each locus was aligned with MAFFT 7.3^62^ with default options (E-INS-i algorithm) and alignments were checked and refined by eyes. Reading frames of coding genes were checked in Mesquite 3.61. The final molecular matrices are made available (Supplementary Data S3). The supermatrix of 15,948 nucleotides was divided into 14 partitions (one per locus), and we identified the best molecular partitioning strategy with PartitionFinder 2.1.1^63^ using the *greedy* search algorithm and the Bayesian Information Criterion.

We simultaneously estimated the phylogenetic tree and divergence times of Carcharhiniformes and Lamniformes using a Bayesian relaxed-clock approach accounting for rate variation across lineages^64^ as implemented in BEAST 1.10.4^65^. We set the following settings and priors: a partitioned dataset (after the best-fitting PartitionFinder scheme) was analysed using the uncorrelated lognormal distribution clock model, with the mean set to a uniform prior between 0 and 1, and an exponential prior (lambda = 0.333) for the standard deviation. The branching process prior was set to a birth–death process^66^, using the following uniform priors: the birth–death mean growth rate ranged between 0 and 10 with a starting value at 0.1, and the birth–death relative death rate ranged between 0 and 2 (starting value = 0.5). Each analysis consisted of four Markov Chain Monte Carlo (MCMC) running for 200 million generations and sampled every 20,000 generations. We performed four independent BEAST analyses to ensure good MCMC mixing and convergence. Convergence and performance of MCMC runs were evaluated using Tracer 1.7.1^67^, along with the effective sample size (ESS, considering ESS > 200 as good convergence) for each parameter (after removing the first 10% generations of each MCMC as burn-in). We also visually assessed the convergence of tree topology. We then combined the four runs using LogCombiner 1.10.4^65^ and reconstructed a maximum-clade credibility (MCC) tree, with posterior probabilities (PP), median age and 95% height posterior density (HPD) for each node, with TreeAnnotator 1.10.4^65^.

We tested the impact of the number of molecular clocks and maximum age for the tree root on the divergence time estimates. Partitioning the molecular clocks has been shown to influence the divergence times^68–70^, and we assessed its impact by comparing dating analyses made with four molecular clocks (three for the mitochondrial markers and one for the nuclear marker) versus dating analyses made with seven molecular clocks (one clock per molecular partition). Likewise, the maximum age of the tree root is known as a key prior for molecular dating^71,72^. We evaluated the effect of maximum age at the root by setting it to 208.5 Ma or 251.9 Ma. The age of 208.5 Ma corresponds to the oldest fossil of Galeomorphii, the shark superorder including Carcharhiniformes, Heterodontiformes, Lamniformes, and Orectolobiformes (see Supplementary Data S4). The age of 251.9 Ma corresponds to a safe maximum age for the divergence between Carcharhiniformes and Lamniformes given that there are no galeomorph fossils in the Permian. In addition, previous molecular estimates of divergence times for carcharhiniforms did not yield ages older than 230 Ma for the divergence between Carcharhiniformes and Lamniformes^43,44^. This resulted in four independent BEAST analyses set either with four or seven molecular clocks and either with a maximum age of 208.5 or 251.9 Ma.

#### Fossil calibrations

Given the extent of the shark fossil record, we selected the oldest definitive fossil occurrence for seven clades following the best practices of Parham et al.^73^. We evaluated the five proposed criteria^73^ to assess the suitability of each fossil calibration (Supplementary Data S4), four of which are fulfilled for each node calibration. However, none of the selected fossil taxa could have their phylogenetic placement assessed within a phylogenetic framework since such a framework is lacking for the vast majority of the tooth-based fossil carcharhiniform species. We relied on an apomorphy-based approach, which requires the phylogeny of extant taxa to be at least partly known^74^. Node calibrations were set using a uniform prior with the minimum age equal to the youngest age of the geological formation where the fossil was found. Additionally, we constrained the divergence between Carcharhiniformes and Lamniformes (= tree root age) with a uniform distribution bounded by a minimum age of 166.1 Ma based on the oldest fossil belonging to these two orders (†*Eypea leesi*^75^) and a maximum age of 208.5 Ma based on the oldest Galeomorphii fossil (†*Reifia minuta*^76^) or 251.9 Ma as a safe maximum age. Absolute ages followed the 2020 International Chronostratigraphic Chart^77^.

#### Extraction of species’ origination and extinction times

**Figure 3:**
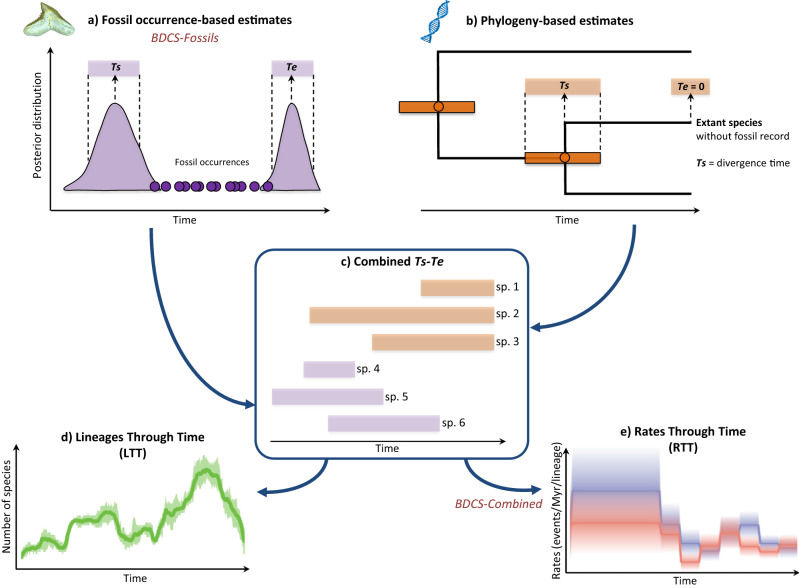
Extraction and combination of inferred times of speciation (*Ts*) and extinction (*Te*). (**a**) Plot showing an example of sampled fossil occurrences (purple circles) with ages obtained from uniform distributions between their minimum and maximum ages (retrieved from the geological strata), and the estimated posterior distributions of *Ts* and *Te* (and 95% HPD). (**b**) Phylogeny showing an example of species relationships with branch lengths proportional to time and nodes representing estimated divergence times (and 95% HPD). (**c**) *Ts* and *Te* estimates for species represented by fossil occurrences are combined with Ts estimates for extant species without fossil record extracted from the phylogeny (*Te* = 0 for extant species). This allows computing the lineages-through-time (LTT) plot (**d**) and the speciation and extinction rates through time (RTT) plot (**e**). Fossil tooth picture of †*Galeocerdo eaglesomei* (Eocene, Togo) from G. Guinot; DNA symbol from Wikimedia Commons.

We extracted from the time-calibrated phylogeny of carcharhiniforms all divergence times leading to extant species. Median ages were obtained for each node using 20 trees randomly taken from the post-burn-in trees distribution of the Bayesian dating analysis (Fig. 3). According to several authors^27,49^, divergence times of extant species are equivalent to the times of species origination (*Ts*), and by definition the time of species extinction (*Te*) is equal to zero for extant species (i.e. not extinct today). This approach allows the inclusion in a single *Ts*–*Te* dataset of all extinct and extant species represented in the fossil record as well as extant species that are not represented in the fossil record. However, it is important to keep in mind that molecular divergence times are not the exact *Ts* because divergence from the sister species does not equate to the origination of the species (see Discussion). Given the known discrepancies between molecular-based and fossil occurrence-based estimates of taxon ages, we assessed the difference between the *Ts* of the 35 extant species that have fossil occurrences but are also present in the dated phylogeny, such that their *Ts* is estimated with both methods. Although there are discrepancies in age estimates as expected, linear regressions with and without two outliers shows positive correlations albeit not significant between fossil occurrence-based and phylogeny-based *Ts* (Supplementary Data S5). In these 35 specific cases, we retained the *Ts* estimated with PyRate.

### Fossil sampling and analyses

#### Fossil occurrences

We compiled species-level taxonomy and fossil occurrence data of carcharhiniform sharks using the data from Condamine et al.^52^, which were updated (July 2020) based on subsequent advances in the literature. We did not consider species left in open nomenclature, except in some rare cases where specimens clearly represented new taxa that were not formally described in the literature (Supplementary Data S6). Each occurrence represents the report of a given fossil species in the literature with unique geographic and stratigraphic origins. Therefore, we did not consider fossil occurrences that are geographically and stratigraphically similar to others already considered (fossils sampled from the same geological horizon in similar locality). This avoids artificially inflating the number of occurrences based on specimens that possibly belong to the same individual or population^52^. Our updated database includes 1397 fossil occurrences of 324 species (Supplementary Data S7), of which 37 are extant species (and 35 have available molecular data).

#### Estimates of fossil occurrence-based diversification rates

We performed Bayesian inferences of the fossil occurrence data under a birth–death model with constrained shifts (BDCS^78^), here referred to as ‘BDCS-Fossils’. The BDCS model estimates the parameters of the preservation process, the times of speciation (*Ts*, i.e. species origination) and extinction (*Te*, i.e. species extinction) of each species, the speciation (λ) and extinction (μ) rates and their variation through time over specific time bins. This method uses all occurences of each fossil species to estimate *Ts* and *Te*, which can be regarded as estimated lifespans of species, while taking into account preservation biases^23^. Hence, species are considered present from their estimated time of speciation until their estimated time of extinction, which produces no Lazarus taxa. We ran PyRate for 10 million of MCMC generations for the BDCS-Fossils analyses with specified time bins (-*fixShift* option). We constrained the rates to be constant within time bins of either 10 Myrs (main analysis) or corresponding to geological epochs (sensitivity analysis). Fossil occurrence-based analyses were set with the best-fit preservation process (*-qShift* option), and also accounted for varying preservation rates across taxa using the Gamma model (*-mG* option), that is, with gamma-distributed rate heterogeneity^23^. We set the standing diversity of the clade (*-N* option set to 284) in constructing the birth–death hyperprior that implicitly corrects for biases due to a possible under-sampling of extant species that have no fossil record. We also ran the BDCS-Fossils analyses without the -*N* option as a sensitivity test. We monitored chain mixing and ESS by examining the log files in Tracer 1.7.1^67^ after excluding the first 10% of the samples as burn-in period. We then combined the posterior estimates of the speciation and extinction rates across all replicates to generate rates-through-time plots of λ, μ, and net diversification rates. Rates of two adjacent intervals (10 Myrs) were considered significantly different when the mean of one lay outside of the 95% HPD of the other, and conversely. We replicated the analyses on 20 randomised data sets and calculated estimates of *Ts* and *Te* as the mean of the posterior samples from each replicate. Thus, we obtained 20 posterior estimates of *Ts* and *Te* for all species and used them to compute the lineages-through-time (LTT) plots.

### Combining neontological and palaeontological data for estimating diversification rates

We estimated the diversification dynamics of carcharhiniforms by adopting an inclusive approach based on both fossil record and phylogenetic data. Combining the fossil occurrence-based and phylogeny-based *Ts*–*Te* (Fig. 3a, b) yielded a global dataset including 483 carcharhiniform species in total (Fig. 3c), and represented by (1) 324 species for which the *Ts*–*Te* are estimated with the fossil record (287 extinct and 37 extant species, of which 35 are sampled in the phylogeny) and (2) 159 species for which the *Ts*–*Te* are estimated with the phylogeny. The combined *Ts–Te* dataset was used to compute a LTT plot based on range-through diversity with the -*ltt 1* option in PyRate (Fig. 3d).

We performed PyRate analyses over the combined dataset using the BDCS model (here referred to as ‘BDCS-Combined’) to estimate speciation, extinction, and net diversification rates (Fig. 3e). Compared to the fossil occurrence data only, these BDCS-Combined analyses relied on the *Ts–Te* as input data, not on fossil occurrence data. Therefore, by using *Ts–Te* data as input, this BDCS-Combined analysis does not allow for re-estimating preservation processes. Furthermore, these analyses did not require to set a best-fit preservation process, nor to account for varying preservation rates across taxa. These were already accounted for in the fossil occurrence-based analyses (BDCS-Fossils), and in the phylogeny-based analyses through the molecular clock rates. However, the diversification parameters (λ and μ) within specified time bins were estimated based on the combined *Ts−Te* data. We took into account the extant standing diversity of the clade (-*N* option set to 284) and also performed sensitivity analyses without this parameter. We ran PyRate for 20 million of MCMC generations estimating speciation and extinction rates to be constant within specified time bins (-*fixShift* option) of 10 Myrs (main analysis) or corresponding to geological epochs (sensitivity analysis). Chain mixing and ESS were examined in Tracer 1.7.1^67^ after excluding the first 10% of the samples as burn-in. We then combined the posterior estimates of the speciation and extinction rates across all replicates to generate rates-through-time plots.

### Tooth crown height data and size classes

Carcharhiniforms gather species with a wide range of sizes and ecologies, and may not show a homogeneous evolutionary history. We used tooth crown size as a proxy for body length and therefore feeding ecological niche (e.g. large teeth correspond to species of high trophic level) based on data gathered in Condamine et al.^52^ for carcharhiniforms and lamniforms. This measure was made following a line running from, and perpendicular to, the crown/root edge up to the apex of the main cusp, in labial view^52,79^. Measurements were made on anterior teeth in order to exclude size differences related to heterodonty (variation in size and shape among teeth of an individual), which is common in shark dentitions. Measurements made on isolated fossil teeth had their position along the jaws determined based on their morphology and knowledge on dentition patterns in both living and extinct elasmobranchs^47^. Because some fossil carcharhiniform species were described on the basis of skeletal remains or incomplete/unillustrated teeth, tooth measurements could not be made for four out of the 287 fossil species (1.4% of missing data). Tooth measurements for extant carcharhiniform species were made for 220 species (77.46%) based on specimens present in the collections of the University of Montpellier or on tooth illustrations in the literature. Crown height values of the 64 remaining extant species were estimated as follows: (1) total body lengths of extant carcharhiniform species were compiled from FishBase^80^ and the literature (Supplementary Data S8); (2) linear regressions of body length versus tooth size were performed for each family, which were all statistically significant (Supplementary Data S9); and (3) equations of linear regressions were used to determine the tooth sizes for extant species with missing data (Supplementary Data S9). All extinct and extant species were assigned to one of the three size classes ("small", "medium" and "large") following the cut-off values provided by Condamine et al.^52^, corresponding to the three main feeding ecologies in today's sharks. The diversification patterns of each size class were analysed separately.

### Exploring putative causes of rate variations

#### Multivariate birth–death

We used the multivariate birth–death (MBD) model to assess to what extent biotic and abiotic factors can explain temporal variation in speciation and extinction rates^53^. Under the MBD model, speciation and extinction rates can change over time (not constant in bins as in the BDCS model) through correlations with time-continuous variables and the strength and sign (positive or negative) of the correlations are jointly estimated for each variable. Hence, rates vary equally across all lineages as in the BDCS model, but can change continuously and not in a piecewise trend with 10-Myrs-bins and constant rate between bins. We applied the model with exponential correlations between speciation/extinction and the selected variables. Hence, the rates are lineage-homogenous, as in the BDCS model. The MBD model replaces clade diversification dynamic with environmental variables, so that the speciation and extinction rates depend on the temporal variations of each factor. The correlation parameters can take negative values indicating negative correlation or positive values for positive correlations. When their value is estimated to be approximately zero, no correlation is estimated. An MCMC algorithm jointly estimates the baseline speciation (λ0) and extinction (μ0) rates and all correlation parameters (*G*λ and *G*μ) using a horseshoe prior to control for over-parameterization and for the potential effects of multiple testing^81^. The horseshoe prior provides an efficient approach to distinguish correlation parameters that should be treated as noise (and therefore shrunk around 0) from those that are significantly different from 0 and represent true signal. Under the horseshoe prior, the prior on the correlation parameters is a normal distribution with mean 0 and variance determined by two hyper-parameters εi and τ. The hyper-parameters εi and τ control the shrinkage (no effect) or release (effect) of each correlation parameter. We applied the MBD model with exponential correlations between speciation/extinction and the selected variables. According to the MBD model, the speciation rates at time t, λ(t) is:

$$\lambda \left(t\right)=\left({\lambda }_{0}\times exp\sum_{i=1}^{N}{G}_{i}{C}_{i}\left(t\right)\right)$$

where λ0 is the baseline speciation rate, where G1, …, GN are the correlation parameters associated with each variable, and C1, …, CN are the environmental variables. The same rationale applies for the extinction rates, μ(t).

We ran the MBD model on the combined *Ts–Te* dataset using 20 million MCMC iterations and sampling every 20,000 to approximate the posterior distribution of all parameters (λ0, μ0, four *G*λ, four *G*μ, and the shrinkage weights of each correlation parameter, ω). We set the -*rmDD* option to 1 in the MBD analyses on the global (order-level) dataset because testing for diversity-dependence in the entire carcharhiniform dataset would not be meaningful considering the wide range of ecologies represented in this order (see above). However, we tested for the effect of diversity-dependence in the MBD analyses per size classes (-*rmDD* 0 option). To identify significant correlations, previous analyses^53^ have relied solely on ω > 0.5, whereas others^52,82^ adopted more conservative selection criteria where correlations were considered strongly significant only when fulfilling the double condition ω > 0.5 and G ≠ 0. When only one of the criteria is fulfilled, this suggests that there is either a weak support (ω < 0.5) for a potential strong effect (95% HPD different from 0) or a strong support (ω > 0.5) for a potential small effect (95% HPD crossing 0). We summarised the results of the MBD analyses by calculating the posterior mean and 95% HPD of all correlation parameters and the mean of the respective shrinkage weights (among 10 replicates), as well as the mean and 95% HPD of the baseline speciation and extinction rates.

#### Palaeoenvironmental variables

We tested for the influence of past environmental variables over the long-term variations in speciation and extinction rates of carcharhiniforms. We focused on five variables that are considered as likely drivers of the evolutionary history of marine metazoan clades^3,7,83,84^, including elasmobranchs^52,85^. Among them, climate change is regularly found to have impacted the evolution of animal diversity^8^. Major trends in global climate change through time are estimated from relative proportions of oxygen isotopes (δ^18^O) in samples of benthic foraminifera shells^86^. We merged deep-sea δ^18^O data from the global temperature curve of Prokoph et al.^87^ that covers the Mesozoic and Zachos et al.^86^ that spans the Cenozoic, and transformed δ^18^O data into deep-sea temperature estimates using the approach of Epstein et al.^88^. While each individual data point is subject to certain biases (e.g. not accounting for sea-level and ice volume fluctuations which are important during periods of large-scale glaciations^89,90^), the interpolated curve smoothens such biases, as well as geographical variations, providing a reliable estimate of global temperature trends^91^. The resulting temperature curves reflect planetary-scale, rather than fine-scale, climatic trends. The index of continental fragmentation developed by Zaffos et al.^84^ was selected as another abiotic variable to represent temporal changes in the geographic arrangement of continental crusts. This index ranges from 0 (maximum tectonic plate aggregation) to 1 (complete plate fragmentation). Sea level data were taken from Miller et al.^92^ and represent eustatic changes due to variations in continental ice sheet volumes, which are recorded by δ^18^O from foraminifera over the Early Jurassic-Recent interval. We also included the δ^13^C data of Prokoph et al.^87^, which is a proxy of oceanic productivity and preservation of organic matter^83^. Finally, it has been proposed that reefs might have acted as a driver of the shark diversity dynamics^44^. We therefore compiled data on biological reef volume variations, i.e. including volume data of rocks bio-constructed by metazoan organisms (e.g. bivalves, sponges, cnidarians, foraminifera), produced by Kiessling and Simpson^93^. Sea-level and δ^13^C data were subsampled every 0.5 Myrs using the *smooth.spline* function in the R-package pspline 1.0–18^94^ with a degree of freedom of 200. All the data are made available (Supplementary Data S10). Among the five selected variables, it could be expected that some covary, especially global temperatures and sea level. However, sea level variations are not only due to temperature change but also due to ice volume, tectonic activity, Milankovitch cycles, and ocean basin dynamics^92,95^. Consequently, most previous macroevolutionary studies that tested the links between these variables and past biodiversity fluctuations found different relationships according to the tested variables^7,52,85^.

## Results

### The carcharhiniform tree of life

We compared our four BEAST molecular datations based on MCMC parameters convergence and congruence with fossils. The analysis, which includes 7 fossil calibrations, 4 molecular clocks and a maximum age for the tree root of 251 Ma, showed the best convergence scores (ESS > 200 for all parameters). Other analyses including a maximum age of 208 Ma or 7 molecular clocks showed a lower convergence threshold, potentially due to a poor prior for the tree root age and/or an unadapted number of molecular clocks. The carcharhiniform tree is well resolved with 66.99% of the nodes having PP⩾0.95 (Fig. 3). On average, the four trees are well supported with 66.74% of the nodes having PP⩾0.95 (see alternative dated trees in Supplementary Data S11). The tree topologies obtained under the four analyses are highly similar and only differ within species-rich and rapidly radiating groups (e.g. *Cephaloscyllium, Hemitriakis,* and *Carcharhinus *sensu lato).

**Figure 4:**
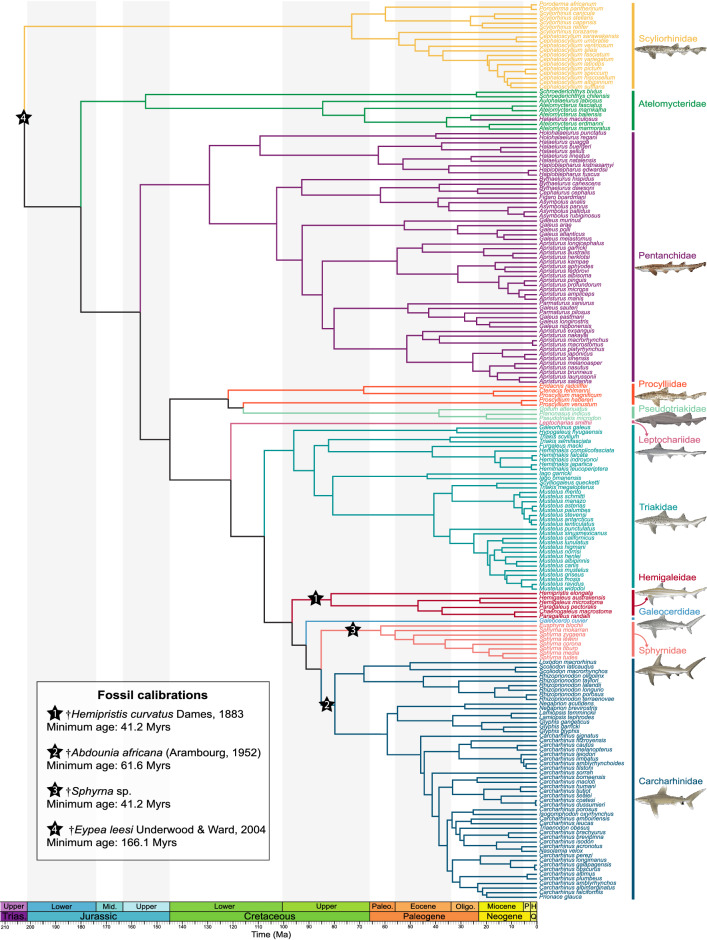
Bayesian time-calibrated phylogeny of Carcharhiniformes and evolution of reproductive strategies and habitats through time. The phylogeny of ground sharks has been reconstructed with 13 mitochondrial protein-coding genes, two mitochondrial RNA genes, and one nuclear protein-coding gene divided into 7 molecular partitions. The divergence times have been estimated using 7 fossil calibrations with uniform priors and 4 molecular clocks (for sensitivity phylogenetic analyses, see Supplementary Data S11). Branches show the carcharhiniform families. Shark images courtesy of Marc Dando (artist).

The divergence time estimates indicate an origin of the crown Carcharhiniformes in the Early Jurassic (191.7 Ma, 95% HPD = 174.7–207.1 Ma) or around the Triassic-Jurassic transition (202.3 Ma, 95% HPD = 187.4–208.5 Ma), depending on the calibrations used (Fig. 4, Supplementary Data S11). All four analyses support the monophyly of the carcharhiniform clade with moderate to maximal support, and all indicate similar interfamilial relationships. Among those, the family Scyliorhinidae (sensu Soares & Mathubara^48^) is monophyletic and recovered as the first offshoot of carcharhiniforms with maximal node support. Atelomycteridae and Pentanchidae (sensu Soares & Mathubara^48^) are recovered respectively as the second and third offshoots of carcharhiniforms with maximal node support. However, the pentanchid species *Halaelurus maculosus* White et al*.*^96^ is nested within the *Atelomycterus* clade, while all other *Halaelurus* species are included in the pentanchids. Considering that the *H. maculosus* sequence analysed was sampled from one of the paratypes of the species (CSIRO H 5890–01), this suggests a need to revise the systematics of this species. The close relationship between the families Proscylliidae and Pseudotriakidae (estimated crown ages between 117.6 Ma, 95% HPD = 89.7–143.0 Ma; and 121.8 Ma, 95% HPD = 94.0–145.9 Ma) is strongly supported (PP > 0.98), these clades being sister to all non-scyliorhinid/atelomycterid/pentanchid carcharhiniforms (PP > 0.99). However, our results do not confirm the monophyly of the proscylliids, some species of the genus *Proscyllium* being more closely-related to Pseudotriakidae than to other proscylliids, but support for the corresponding node is weak in the four analyses (PP < 0.83). The monospecific family Leptochariidae is recovered sister to all more derived carcharhiniforms, and diverged in the Early Cretaceous (between 117.8 Ma, 95% HPD = 101.2–135.2 Ma; and 120.8 Ma, 95% HPD = 106.8–135.6 Ma). The families Triakidae, Hemigaleidae, and Sphyrnidae are found monophyletic with maximal node support for the latter two and moderate support (> 0.93) for triakids. Node age estimates indicate a Late Cretaceous origin for these clades. The species *Galeocerdo cuvier* is consistently found out of the Carcharhinidae, sister to a clade including Sphyrnidae and Carcharhinidae (PP = 1), which supports its position in its own family (Galeocerdidae; see Ebert et al.^42^). The monophyly of Carcharhinidae (excluding *G. cuvier*) received maximal support and the origins of the clade are estimated in the Late Cretaceous, between 77.9 Ma (95% HPD = 67.4–87.9 Ma) and 79.7 Ma (95% HPD = 70.4–89.2 Ma).

### Diversification and diversity dynamics through time

**Figure 5:**
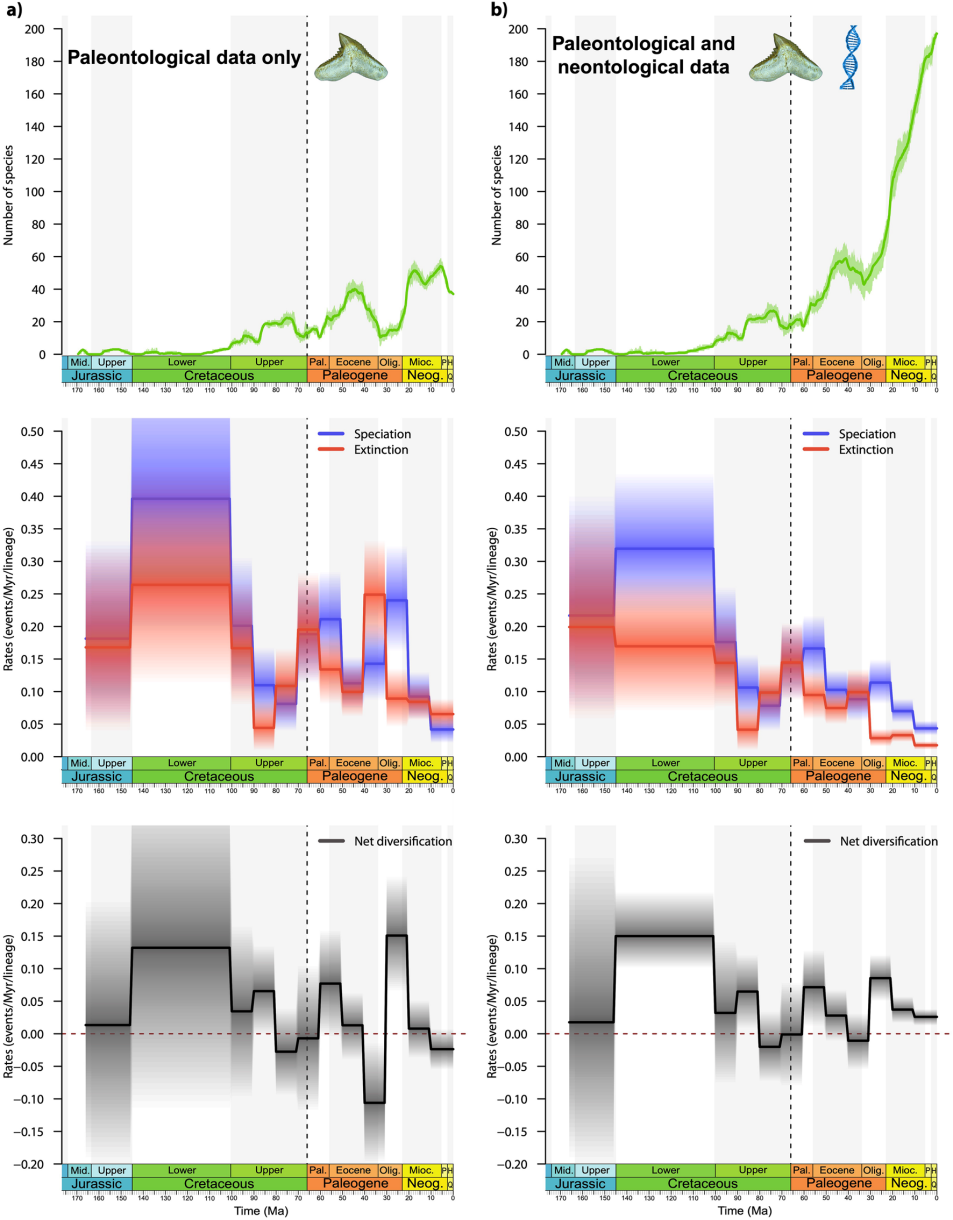
Diversification dynamics of Carcharhiniformes estimated with (**a**) the fossil occurrence dataset only (BDCS-Fossils), and with (**b**) a combination of the fossil and phylogenetic datasets (BDCS-Combined). The birth–death model with constrained shifts (BDCS, shifts every 10 Myrs) was used to infer speciation (blue) and extinction (red) rates and their temporal variation. The net diversification rates (black) are the difference between speciation and extinction rates (rates below 0 indicate declining diversity). Solid lines indicate mean posterior rates and the shaded areas show 95% HPD. For the fossil occurrence-based analyses, the BDCS-Fossils infers negative net diversification rates that lead to a diversity decline for the group at the Cretaceous-Paleogene and Eocene–Oligocene boundaries, and also in the mid-Miocene and the last 5 million years. On the contrary, for the combined fossil-phylogenetic dataset, the BDCS-Combined does not indicate such negative rates in the mid-Miocene and the last 5 million years. Instead, the group expands rapidly from the Eocene–Oligocene boundary onward (for sensitivity analyses with the BDCS-Combined, see Supplementary Data S13, S15).

The fossil record of carcharhiniforms includes 1,397 occurrences assigned at the species level, which represent 324 species (37 extant and 287 extinct). We inferred the diversification history of carcharhiniforms using fossil data only through a birth–death model (BDCS-Fossils). Shifts were analysed using predefined time intervals (10 Myrs or geological epochs), including or not the -*N* parameter informing the number of extant species, where rates can change between time bins (Supplementary Data S12–S13). Results of the BDCS-Fossils analyses without the -*N* option provided similar rate results as the analysis using the -*N* option (Supplementary Data S13), indicating no effect of this option in correcting for missing extant species in the fossil occurrences dataset. Regardless of these analytical settings, our results indicate that the diversity dynamics of carcharhiniforms conform to a time-variable birth–death process characterised by low diversity in the Mesozoic and increased diversity in the Cenozoic (Fig. 5; Supplementary Data S13). The diversification pattern is marked by high background extinction rates and is punctuated by peaks of extinction rates preceding the K-Pg boundary and at the EOT, followed by high speciation rates in the aftermath of these two events. Both speciation and extinction rates decrease toward the present with extinction being higher than speciation in the last 10 Myrs, indicative of a diversity decline.

**Figure 6:**
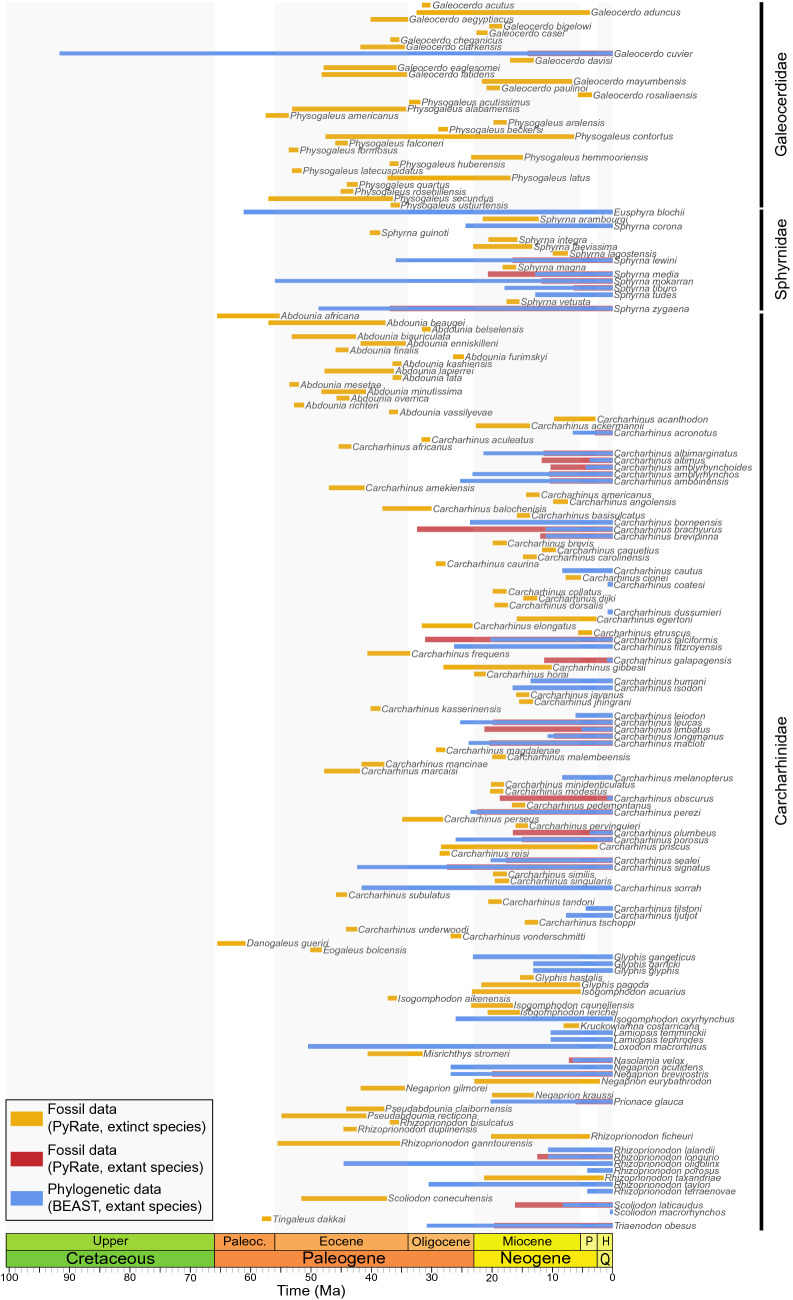
Estimates of species lifespan from the fossil record and the molecular phylogeny for the clade gathering Galeocerdidae, Sphyrnidae, and Carcharhinidae. Species lifespan is determined by the times of speciation (*Ts*) and times of extinction (*Te*), as inferred with the fossil record for extinct species (yellow) and extant species having fossil occurrences (orange), and those inferred with the dated phylogeny for extant species only (blue). Summing up the number of species per million-year bins allows recovering the diversity dynamics of the clade through time. See Supplementary Data S14 for data on the entire Carcharhiniformes.

After combining fossil occurrence-based and phylogeny-based *Ts*–*Te* data (Fig. 6; Supplementary Data S14), the BDCS-Combined analysis also reveals a time-dependent diversification pattern that is very similar to the pattern inferred with fossil data only until the early Miocene, although the rate magnitude differs from the middle Eocene onward (Fig. 5; Supplementary Data S15). Combined data indicate two periods of negative net diversification rates at 70–80 Ma and at the EOT, both due to a joint effect of higher extinction and lower speciation rates. However, diversification patterns including neontological data diverge from those based on fossil data only over the last 20 Myrs (even when the diversity through time is log-transformed; Supplementary Data S16). Combined data indicate a speciation burst from the early Miocene onward, which is in line with the high Oligocene speciation rate, while fossil data indicate decreasing speciation rates, which results in negative net diversification in the last 10 Myrs (Supplementary Data S16).

**Figure 7:**
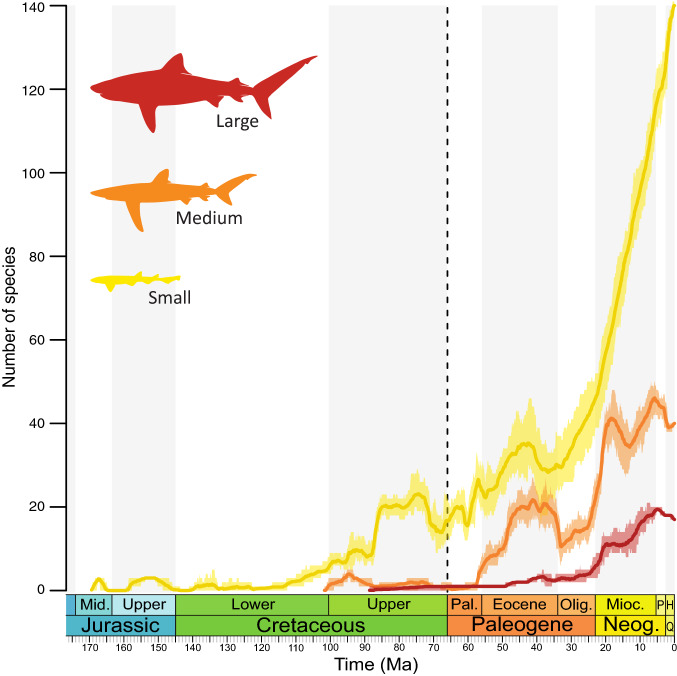
Variations in carcharhiniform species richness through time by size classes, inferred with birth–death model with constrained shifts (BDCS-Combined, shifts every 10 Myrs) using combined fossil and phylogeny datasets. Solid lines indicate mean diversity and the shaded areas show 95% HPD. Complete rate estimates (extinction, speciation, and net diversification) for each size class are available in Supplementary Data S17.

We also estimated the diversification dynamics of three carcharhiniform classes (based on tooth size akin to ecological niches) with the combined fossil and phylogenetic dataset under the BDCS model (shifts every 10 Myrs, Supplementary Data S17). The results show a trait-specific pattern of diversification with different temporal origins (Fig. 7). The small-sized carcharhiniforms have an old (Late Jurassic) origin, low diversity throughout the Mesozoic, are little impacted by the K-Pg event, and show a delayed explosive radiation since the EOT onward. The medium-sized carcharhiniforms have an intermediate (mid-Cretaceous) origin, with a post-K-Pg diversification punctuated by the EOT, but followed by a post-EOT recovery and a strong diversification in the Miocene that levels off toward the present. The large-sized carcharhiniforms have a younger (Late Cretaceous) origin, with a steady diversification toward the present.

### Drivers of the carcharhiniform diversity patterns

**Figure 8:**
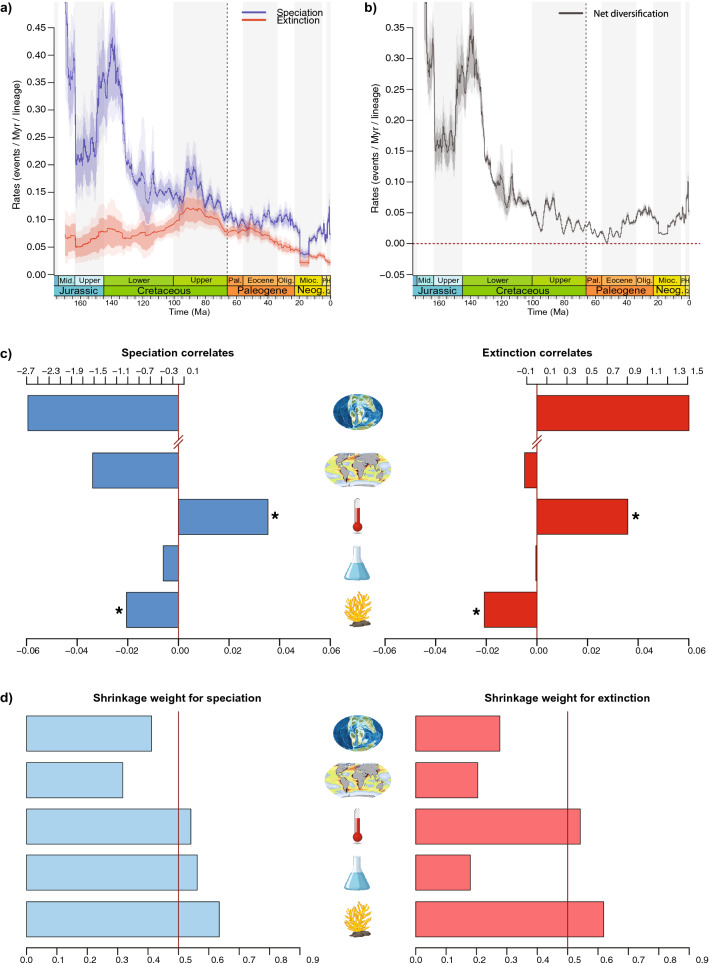
Dynamics of rates through time estimated with the Bayesian Multivariate Birth–Death model. Using the combined palaeontological and neontological data, we incorporated the effect of five environmental factors over speciation and extinction (**a**), and net diversification (**b**) for carcharhiniform species. Solid lines indicate mean posterior rates and shaded areas show 95% HPD. The contribution of each environmental variable to the dynamics of rates through time is represented by Bayesian inferences of correlation parameters on speciation (blue bars) and extinction (red bars) with the tested environmental parameters (**c**) and shrinkage weights (**d**). Highly significant correlations are represented by an asterisk and ω > 0.5 (red vertical bar). For details on the estimations of parameter correlates, see Supplementary Data S18. In panels (**c**) and (**d**), icons represent the five environmental variables, which are from top to bottom: continental fragmentation (index), oceanic productivity (δ13C in ‰), temperature (in °C), sea level (in metres), and biological reef volume (in km^3^). In panel (**c**), the upper axis refers to correlates for the continental fragmentation index, whereas the lower axis refers to all other variables, due to scale differences.

The MBD analysis relying on fossil and phylogenetic data shows a combined effect of all variables (Fig. 8; Supplementary Data S18), with a heterogeneous contribution of each variable. Two variables fulfilled the selection criteria of significance: (1) a positive effect of global temperature on both speciation (*G*λ = 0.0354, ω = 0.5411) and extinction (*G*μ = 0.0359, ω = 0.5418), suggesting higher speciation and extinction rates during warmer periods; (2) a negative effect of reef volume on both speciation (*G*λ = − 0.0205, ω = 0.6346) and extinction (*G*μ = − 0.0208, ω = 0.6183), suggesting lower speciation and extinction rates during periods of increased reef construction (Fig. 8). The MBD analysis based on fossil data only indicates a significant negative correlation between reef volume and speciation (*G*λ = − 0.0253, ω = 0.647; Supplementary Data S18).

The MBD analyses per size class indicate multiple effects of the tested variables (Supplementary Data S19), with two variables being strongly supported by the selection criteria. For small carcharhiniforms, results indicate: (1) a negative diversity-dependence on extinction (*G*μ = − 2.9997, ω = 0.8869), suggesting that extinction decreased as the diversity of small carcharhiniforms increased; (2) a negative correlation between reef volume and extinction (*G*μ = − 0.0533, ω = 0.8834), suggesting that extinction decreased as reefs expanded. Furthermore, results for other size classes suggest a marked effect of negative diversity dependence on speciation for medium (*G*λ = − 1.8742, ω = 0.746) and large (*G*λ = − 1.8996, ω = 0.7533) carcharhiniforms. This indicates a strong support but a weak effect of negative diversity dependence in these ecological groups.

## Discussion

Studying macroevolutionary history has long relied either on the fossil record or on molecular phylogenies separately. Although there have been calls to integrate both types of data^25,26^, especially when estimating extinction rates^17^, studies combining palaeontological and neontological data remain scarce. Models allowing the estimation of diversification rates from such an integrative framework are in their infancy, notably for making use of total-evidence dated phylogenies^34^, and these models do not allow to assess the role of abiotic or biotic variables on diversification yet. In addition, tree-based approaches are challenging for most groups because both molecular data (for extant species) and morphological data (for extant and extinct taxa) are needed, which requires a considerable amount of time and comes with issues. For instance, the dynamics of carcharhiniform rates of diversification and diversity could have been analysed with the occurrence birth–death process^33^. However, the lack of existing morphological character matrix hinders the placement of fossil carcharhiniform species in the tree, and the clade size makes the analyses computationally intensive (J. Andréoletti pers. com.). The only study using total-evidence dating for an elasmobranch group focused on the shark order Squaliformes that contains ~ 140 extant species and ~ 74 extinct species, of which 17 extant species have a fossil record^97^. Although this attempt has merits, the resulting total-evidence tree only includes 32 extant species (~ 23% of the total diversity) and 31 extinct species. Considering the highly incomplete taxon sampling, such a time-tree would likely provide uncertain estimates of diversification rates^13^. Tree-based approaches probably represent future avenues of research in macroevolution, but other approaches that break free from a total-evidence framework should be considered.

We studied the most speciose shark order, for which a total-evidence dating would be very challenging as no matrix gathering morphological characters can be used for integrating fossils in an extant time-tree. Such a dataset is extremely complicated to assemble because of the high diversity of dental forms that are probably related to the broad ecological habits and feeding strategies, which makes the definition of shared morphological characters and identification of homologous structures difficult. Therefore, we have built an approach relying on both the fossil occurrences and a time-calibrated molecular phylogeny to estimate times of speciation (*Ts*) and of extinction (*Te*) from the fossil data for both extinct and extant species (for those that have a fossil record), and *Ts* from the phylogeny of extant species (*Te* for extant species being equal to zero). These *Ts* and *Te* were then combined in a single dataset analysed with PyRate using only the *Ts* and *Te*^53,78^.

This methodology has some advantages for macroevolutionary studies. First, this is relatively easy to implement, although there are still some difficulties in assembling a molecular phylogenetic dataset along with a fossil dataset at the same taxonomic level. Nonetheless, time-calibrated phylogenies are becoming increasingly popular, even for non-model clades, and there are online fossil databases (e.g. *Paleobiology Database*) that gather a massive number of fossil occurrences for some groups (e.g. mammals, bivalves). Second, our approach is particularly appropriate for clades that experienced recent radiations but lack a fossil record for most living species, like shown with the Carcharhiniformes. If the *Ts* cannot be estimated directly from the fossil data for some extant species, molecular divergence times of these species can be recovered from phylogenies. This provides a better sampling of the extant diversity of the focal group, and a better estimation of diversification rates in the recent past. Third, combining *Ts* and *Te* for all sampled species is made while taking into account age uncertainties either through the fossil preservation rate, or the molecular clock rate. These *Ts* and *Te* represent the lifespan of each species in the group, hence allowing to estimate the temporal diversity dynamics. Fourth, the diversification analyses are performed in a Bayesian framework that allows jointly estimating speciation, extinction, preservation rates, and the rate shifts as well as the magnitude of these rates without phylogeny^23,24,78^. Fifth, this approach would theoretically provide more accurate speciation and extinction rates for the focal group, in particular extinction rates that are often seen as poorly estimated with phylogenies of extant species only^12,17^ (but see Morlon et al.^98^).

Both phylogeny-based and fossil occurrence-based analyses converged on a crown age between 208 and 170 Ma for the carcharhiniforms. This is in line with previous molecular dating proposed by Sorenson et al. (2014), which suggested a crown age of 179 Ma (95% HPD = 165–199 Ma). Carcharhiniform diversification patterns obtained through fossil occurrence-based analyses and combined fossil and molecular data are consistent over most of the evolutionary history of the clade and only differ in the dynamics through the early Miocene-Recent interval (Fig. 5; Supplementary Data S16). Our results indicate a complex evolutionary history marked by a high variability in net diversification rates, with a succession of ups and downs throughout the entire lifespan of the group. This is followed by a strong diversification burst resulting from a drop in extinction coupled with high speciation rates over the last 30 Myrs. This suggests a decreasing fossil record quality across the Miocene-Recent interval, which seems to prevail for most elasmobranch clades at low taxonomic levels^51^. This is likely related to a combination of lower sampling and lack of knowledge on the anatomy of some modern elasmobranch clades, and upholds the combination of phylogenetic and fossil record data developed here. Identifying remains of some living elasmobranch species in the fossil record is difficult due to incomplete knowledge on the tooth morphology of many living species^41^, especially among carcharhiniforms. This may also lead to underestimating species-level diversity in the recent past. Furthermore, Pliocene elasmobranch faunas are comparatively less studied than older fossil assemblages. The underrepresentation of outcropping post Miocene-Pliocene offshore and deep-water rocks may contribute to the observed Miocene-Recent drop, since numerous carcharhiniform taxa (especially pentanchids, pseudotriakids, and some scyliohrinids) dwell in these environments^42^. Our dated phylogeny indicates increasing cladogenesis from the Eocene onward for these clades, which is supported by the fossil record. Deep-sea fossil assemblages contain no or rare carcharhiniforms in the Cretaceous^99–102^, whereas this clade is well represented in post-Paleocene deep-sea assemblages^103–105^. Hence, colonisation of deepwater environments likely promoted the late burst of carcharhiniform diversification through ecological opportunity, as previously hypothesised^44^. Interestingly, other speciose chondrichthyan orders (squaliforms, rajiforms) encompass a large proportion of species adapted to deepwater and it is possible that this environment played an important role in elasmobranch radiations. The macroevolution of carcharhiniforms complies with a two-step pattern with the first part of their evolutionary history (Jurassic and Early Cretaceous, 170–100 Ma) consisting of a low-diversity period with high turnovers, while the second part (Late Cretaceous onward) is characterised by a radiation that is exacerbated in the aftermath of the EOT (Fig. 5; Supplementary Data S16). Our diversification analyses provide clues to explain the temporal decoupling of diversification.

Both the dated phylogeny and fossil record indicate that the low diversity estimated for more than 70 Myrs in the early history of the group is mainly represented by small-sized scyliorhinid-like clades^50^, while the diversification of other clades and ecologies did not occur before the Late Cretaceous and early Cenozoic (Figs. 4, 7). Nonetheless, this low-diversity period could potentially be impacted by biases related to fossil data. The elasmobranch fossil record in these periods is strongly dominated by assemblages from European localities, and samplings from other regions might modify the observed pattern. This is consistent with the high number of genus-level ghost lineages inferred for elasmobranchs during this time interval^50^, suggesting incomplete sampling. However, additional data from undersampled regions would hardly reverse the pattern observed given the strength of the difference in palaeobiodiversity between pre and post mid-Cretaceous time intervals.

In contrast with the Jurassic-Early Cretaceous diversity, the Late Cretaceous-Recent diversification complies with an expansion (unbounded) model, which is only interrupted by two periods of declining diversity represented by negative net diversification rates: around the K-Pg boundary and the EOT. Interestingly, the first diversity drop occurs in the Campanian (~ 75 Ma), well before the K-Pg extinction and is the result of low speciation rates. This period is known to correspond to climatic and oceanic perturbations, including a marked cooling^106,107^. Our analyses indicate that the K-Pg boundary actually corresponds to a phase of increasing diversity with high turnover and shows no evidence of marked extinction event. This agrees with the hypothesis of a strong phylogenetic selectivity of the K-Pg event among elasmobranchs, with the carcharhiniforms being among the less affected clades^50^. Arguments for such a phylogenetic selectivity can also be found in our time-calibrated phylogeny where 30 carcharhiniform lineages cross the K-Pg boundary (Supplementary Data S11, S14). The effect of the EOT on marine vertebrates has not been studied in detail, but recent analysis of the lamniform sharks indicated that this event had a strong impact over their evolution, especially large-sized species^52^. Our results provide additional evidence that the EOT and preceding late Eocene fostered elevated extinctions in some marine vertebrates. This mirrors the marked extinctions reported at or near the EOT for marine invertebrates and terrestrial vertebrates, which were linked with major global cooling^108,109^.

Studying the putative drivers of diversification represents a fascinating but challenging topic^6,52^. This is especially true for ancient groups whose diversification dynamic is often complex and is likely the result of multiple intertwined drivers that vary through time and do not impact the group homogeneously. Our MBD analyses provide insights into the factors likely leading to variations of speciation and extinction through time and across ecological classes, although we should keep in mind that we did not assess the effect of all possible drivers. At the order level, our results indicate that the tested environmental variables all played a combined role in the observed diversification pattern, with two main environmental correlates exerting significant evolutionary pressure on the clade’s diversification (Fig. 8). Our analyses pinpoint periods of climate change and variations in reef volume as likely drivers of diversification. Both speciation and extinction rates are found to depend positively on global temperature, suggesting higher speciation and extinction rates during warm periods and conversely. This result is in line with the metabolic theory of biodiversity predicting a positive link between speciation and temperature^8,110^. Although the role of temperature on extinction is less appreciated at the macroevolutionary scale, we find that the long-term global cooling led to lower extinction in carcharhiniforms toward the present. Interestingly, this is an opposite pattern to their sister order, the Lamniformes, for which global climate cooling was shown to have favoured extinction^52^. Warm periods led to high speciation and extinction rates among carcharhiniform species, which can partly explain the high turnover in the Jurassic-Cretaceous interval estimated with the BDCS analyses. We further show that the expansion of biological reefs correlates with both lower speciation and extinction rates, suggesting a role of museum of diversity (low extinction) but not of a cradle of diversity (high speciation). Our results further refine knowledge on the drivers of carcharhiniforms diversification, which was hypothesised to be linked with reefs, in particular for Carcharhinidae, with higher speciation and multiple independent colonisations of reef ecosystems since the Late Cretaceous^44^. Previous analysis of environmental controls over the genus-level elasmobranch diversification patterns found a positive relationship between diversity and variations in continental fragmentation and sea-level fluctuations^85^, but did not test for the effect of variations in reef expansion. These results and previous work on lamniform sharks^52^ suggest that different elasmobranch clades responded heterogeneously to past environmental change. Carcharhiniforms probably represent a particular elasmobranch clade as attested by their recent diversification burst, which contrasts with the globally decreasing elasmobranch palaeobiodiversity since the Paleogene^50^.

Our analyses also support heterogeneous carcharhiniform diversification dynamics across ecological classes (Supplementary Data S19), with different responses to the environmental proxies tested. The MBD results indicate that the diversification of small carcharhiniforms was affected by several factors, among which reef expansion played a significant role in decreasing extinction (museum of diversity). Although reef-association is not the main ecology among living small carcharhiniforms, the fossil record suggests that this ecology was more largely represented in scyliorhinid-like and triakid carcharhiniforms in the Mesozoic^75,111^ and Cenozoic^112,113^. This tends to indicate a decreasing proportion of reef-associated small carcharhiniforms among fossil faunas towards the present compared with shelf and deep-sea environments. The role of global temperature is not recovered for any ecological classes, which suggests it has a global effect rather than an ecological- or clade-specific impact. The tested environmental variables are not primary drivers of the diversification of large and medium-sized carcharhiniforms at global scale. Overall, our analyses indicate that environmental parameters alone do not entirely explain the complex diversification pattern observed for carcharhiniforms across their evolutionary history. Importantly, the MBD analyses show evidence of a negative diversity-dependence on extinction for small carcharhiniforms and a potential weak effect of negative diversity dependence on speciation for all three ecological classes of carcharhiniforms. This is in line with the globally increasing taxonomic diversity of these ecological groups in the recent past, especially small carcharhiniforms, and is supported by evidence of niche partitioning observed in living members of this order^114–116^.

Our study combining independent datasets is based on a few assumptions and can produce several limitations. Our approach relies on phylogeny-based and fossil occurrence-based estimates of species’ lifespans (*Ts–Te*), which are based on a number of well-known model assumptions pertaining to molecular relaxed clocks^64,71^ as well as for PyRate birth–death models^23,24^. In particular, the latter models assume that (1) speciation to occur by budding, bifurcating and anagenesis; (2) extinction occurs at the end of a taxon lifespan and can happen following anagenetic and bifurcating speciation events (in these cases, the ancestral species goes extinct), (3) both speciation and extinction rates are homogeneous across lineages; (4) preservation can vary through time; (5) preservation is heterogeneous across taxa. However, combining *Ts–Te*, as proposed here, comes with the main assumptions that (1) rates are homogeneous across lineages of the studied clades and that (2) taxon sampling is random, whereas both fossil occurrences and phylogenetic samplings are known to be non-random^3,11,13,22–24^. Nonetheless, this can be circumvented by analysing clade subsets, as done here with size class analyses.

Although the combination step of our approach has not been tested for statistical performance through simulations, we acknowledge this study involves several limitations that also apply to most approaches, including tree-based methods. First, it must be borne in mind that biological and phenotypic species are not necessarily similar, and combining fossil (phenotypic) and living (biological or phylogenetic) species might result in the inclusion of heterogeneous taxonomic levels. However, although different species concepts are used in palaeontology and neontology, palaeontological and neontological approaches for species delimitations are similar, and today most fossil species are probably in line with the biological species concept^117^. This is particularly true for sharks whose tooth morphology represents the main arguments for fossil taxonomy, but is also useful for species delimitations in extant taxa^47,118–120^. Second, the sampling fraction of extinct species is probably lower than that of living relatives. Taking both data sets together might tend to increase the sampling contrast between living and fossil species and may artificially increase speciation in recent times (‘pull of the Recent’). Such a discrepancy between fossil- and phylogeny-based rates of diversification can be assessed using models for testing compatibility between both estimates, such as the birth–death chronospecies (BDC) model^121^. In our case, the BDC model supports compatible rates of diversification between fossil and phylogenetic datasets, which indicates that the patterns of diversification provided by the combination of both datasets are congruent (Supplementary Data S20). Therefore, although the sampling fraction between phylogeny and fossil record data are probably heterogeneous, they provide comparable diversification patterns.

Despite these limitations, this approach is relevant for clades with a poor fossil record in recent times combined with recent diversification events that are not entirely captured by the fossil record. Although methods for correcting *Ts–Te* of sampled taxa have been developed, one of the main challenges in palaeodiversity estimates is the inference of non-sampled taxa^33,122^. This is particularly problematic for clades like Carcharhiniformes, for which lineages from some environments (deep-sea) and periods of time (Oligocene, upper Miocene-Recent) are weakly sampled. Among these developments, the mcmcDivE method in PyRate^122^ aims at computing corrected palaeodiversity estimates that account for non-sampled lineages, while incorporating the number of extant species and the effect of temporal variations of preservation rates. However, applied to Carcharhiniformes, mcmcDivE produces comparable trends to the BDCS-Fossils estimates (Supplementary Data S21) and has little effect in the correction of the carcharhiniform palaeodiversity pattern. When compared to the BDCS-Combined estimates, the BDCS-Fossils and mcmcDivE estimates fail to capture the recent diversification pattern of this group and corresponding non-sampled lineages. This further supports the use of alternative approaches for clades experiencing rapid diversifications with an undersampled fossil record in recent times and in some environments. This situation is exemplified by carcharhiniforms since fossil occurrence-based estimates of rates through time show a drop in diversification since 20 Ma (Fig. 5a). This departs from the strong diversification initiated in the Oligocene, which is in line with the tremendous diversity of living species. The fossil occurrence-based species-level diversification pattern is opposite to the ‘pull of the Recent’ for this clade, and the limited fossil record for the recent past clearly requires to complement fossil data by the addition of neontological data^41^. Hence, adding the *Ts* of living species produces more realistic diversification patterns, although the amplitude of the past diversity fluctuations might not be homogeneous between the recent past (dominated by taxa with *Ts* extracted from phylogeny) and older time periods (dominated by fossil data). However, our approach tends to overestimate *Ts* for some taxa with long branches (e.g. *Leptocharias smithii*) or undersampled clades, due to incomplete sampling of living taxa in the phylogeny that reduces the chances of splitting recent lineages (cladogenetic events overlooked) and tends to provide artificially old *Ts*. For example, the genus *Parmaturus* is represented by two species in the phylogeny, while containing nine living species. Sampling of the remaining seven species could result in shortening branch lengths of members of the clade, and provide younger *Ts* (similar examples with *Bythaelurus*: 3/14, *Galeus*: 9/18, or *Cephaloscyllium*: 12/21). This can counterbalance the strong recent diversification related to the addition of living taxa. However, adding extant taxa does not necessarily produce young *Ts*. The comparison between *Ts* estimated with fossils and phylogeny for the 35 extant species represented in both data (Supplementary Data S5) shows a substantial number of instances (21/35) where the phylogeny produces slightly older ages than the fossil record, even for well-sampled clades (e.g. *Carcharhinus*). It is expected that phylogenetic estimates produce older *Ts* than fossil occurrence-based estimates because *Ts* from phylogenies do not exactly reflect speciation age, but instead divergence time from the sister species (Fig. 3). Furthermore, the mean difference between *Ts* estimated from the phylogeny and *Ts* from the fossil record is weak and positive (5.67 Ma) and even weaker (2.33 Ma) when two outliers are removed (Supplementary Data S5). Consequently, adding *Ts* data of living species extracted from the phylogeny does not necessarily mean adding young species in the dataset, and the pattern of recent diversification burst observed in carcharhiniforms is likely realistic and in line with the fossil occurrence-based diversification observed prior to the Miocene and with the observed diversity of living species. However, although adding *Ts* data of living species helps provide a more complete picture of the speciation pattern, it does not impact extinction rates, which remain driven by fossil data (only the magnitude of extinction rates is adjusted). The pattern of extinction provided by fossil data is thus preserved.

## Conclusions

We estimated deep-time diversity dynamics by combining species-level palaeontological and neontological data in a Bayesian framework. Taking the carcharhiniform sharks as a model group, we estimated times of speciation and extinction from fossil occurrences analysed through process-based birth–death models, which were complemented by times of speciation from time-calibrated molecular phylogenies for living species that are not represented in the fossil record. Bayesian inferences of the combined dataset yielded estimates of variations in speciation and extinction rates as well as diversity fluctuations over the entire clade’s evolutionary history. Our approach supports (1) a complex evolutionary history exemplified by the numerous variations in diversification rates through the ~ 180 Ma of the clade’s lifespan with an early low diversity period followed by a radiation exacerbated since 30 Ma, and (2) the role of reef expansion and temperature change to explain such variations in diversification through time. Our study also highlights the benefits of combining fossil and phylogenetic data to address macroevolutionary questions. This approach is particularly suited for clades with limited fossil record in recent times, especially when coupled with recent and rapid diversification events. It also has advantages in that it does not require a phylogenetic framework for fossil data.

## Supplementary Information

Supplementary Information 1.

Supplementary Information 2.

Supplementary Information 3.

Supplementary Information 4.

Supplementary Information 5.

Supplementary Information 6.

Supplementary Information 7.

Supplementary Information 8.

Supplementary Information 9.

Supplementary Information 10.

Supplementary Information 11.

Supplementary Information 12.

Supplementary Information 13.

Supplementary Information 14.

Supplementary Information 15.

Supplementary Information 16.

Supplementary Information 17.

Supplementary Information 18.

Supplementary Information 19.

Supplementary Information 20.

Supplementary Information 21.

## Data Availability

All data generated or analysed during this study are included in this published article (and its Supplementary Information files).
