# Supplementary material for: Combining palaeontological and neontological data shows a delayed diversification burst of carcharhiniform sharks likely mediated by environmental change
Source: Sci Rep. 2022 Dec 19;12:21906. doi: 10.1038/s41598-022-26010-7 (PMC9763247; doi:10.1038/s41598-022-26010-7)
Supplement: Supplementary file 4 — Supplementary Information 4. [file 41598_2022_26010_MOESM4_ESM.pdf]

Supplementary Data S4. Detailed information of the seven fossil calibrations and justifications following best practices of Parham et al. (2012). For each fossil taxon, information on specimens, justifications on phylogenetic assignment, ages of geological formations are reported for the molecular dating analyses. CR = criterion for best practices *sensu* Parham et al. (2012), MRCA = most recent common ancestor, Myrs = million years.

Fossil taxon #1: †*Hemipristis curvatus* Dames, 1883

- **Node calibrated:** Crown node of family Hemigaleidae.
- **Node (see Fig. 4):** MRCA of *Hemipristis elongata* to *Paragaleus randalli*.
- **Reference specimens (CR #1):** two specimens reported by Underwood et al. (2011), deposited in the Cairo Geological Museum (prefix CGM). The specimens were sampled from the Midawara Formation at Wadi Al-Hitan, Fayum area, Egypt.
- **Phylogenetic placement (CR #2 and CR #3):** Fossils unequivocally assigned to the family Hemigaleidae and to the Recent genus *Hemipristis* on the basis of a combination of several morphological (dental) characters (Cappetta, 2012). A formal phylogenetic position of this species has not been established by morphological characters.
- **Minimum age:** 41.2 Myrs (Cohen et al. 2013).
- **Locality, stratigraphic and age information (CR #4 and CR #5):** These specimens were sampled from glauconitic levels within the Midawara Fm. at the top of which the transition between nannoplankton zones 16 and 17 occurs (Strougo, 2008) and therefore, the age of the Lutetian/Bartonian boundary is used as the youngest age estimated for this calibration.
- **Previous use(s) as calibration and Discussion:** Not used in any previous dating analysis.
- **Best practices score:** 4/5.

References

Cohen, K.M., Finney, S.C., Gibbard, P.L. & Fan, J.-X. 2013 (updated 2020). The ICS International Chronostratigraphic Chart. *Episodes* 36: 199-204.

Dames, W.B. 1883. Über eine tertiäre Wirbelthierfauna von der westlichen Insel des Birket-el-Qurun im Fajum (Aegypten). *Sitzungsberichte der Königlich Preussischen Akademie der Wissenschaften zu Berlin* VI: 129–153.

Strougo, A., 2008. The Mokattamian Stage: 125 years later. M.E.R.C. Ain Shams University. Earth Science Series 22, 47–108.

Underwood, C.J. and Ward, D.J. 2011. New hemigaleid shark from the late Eocene of Wadi Al-Hitan, Egypt. *Journal of Vertebrate Paleontology* 31 (3): 707–711.

Fossil taxon #2: †*Abdounia africana* (Arambourg, 1952)

- **Node calibrated:** Crown node of family Carcharhinidae.
- **Node (see Fig. 4):** MRCA of Carcharhinidae *sensu stricto* (from *Loxodon macrorhinus* to *Nasolamia velox*).
- **Reference specimens (CR #1):** several specimens reported by Noubhani & Cappetta (1997), deposited in the collections of the University of Montpellier, France. The specimens (isolated teeth) were sampled from ImínTanout (Meskala Basin) and Youssoufia (Ganntour Basin), Morocco.
- **Phylogenetic placement (CR #2 and CR #3):** Fossils assigned to the family Carcharhinidae on the basis of a combination of several morphological (dental) characters (Cappetta, 2012; Noubhani & Cappetta, 1997). A formal phylogenetic position of this species has not been established by morphological characters. The species *Archaeogaleus lengadocensis* Guinot, Cappetta & Adnet, 2014 was described as the oldest Carcharhinidae, but it more likely belongs to a stem-group carcharhinoid.
- **Minimum age:** 61.6 Myrs (Cohen et al. 2013).
- **Locality, stratigraphic and age information (CR #4 and CR #5):** These specimens were sampled from phosphorite deposits of Morocco levels. No formal geological formations have been defined in this area and the biostratigraphic framework is mainly based on elasmobranch assemblages, calibrated by chemiostratigraphic data from adjacent basins (Yans et al. 2013, Kocsis et al 2013). This indicates a Danian age for the levels P5 of the Meskala Basin (ImínTanout) and Couche 0 of the Ganntour Basin (Youssoufia). The Danian stage spans the time between 66 and 61.6 Myrs ago (Cohen et al. 2013). Therefore, we used the youngest age estimated for this stage for this calibration (61.6 Myrs).
- **Previous use(s) as calibration and Discussion:** Not used in any previous dating analysis.
- **Best practices score:** 4/5.

References

Arambourg, C. 1952. Les vertébrés fossiles des gisements de phosphates (Maroc-Algérie-Tunisie). *Notes et Mémoires du Service géologique du Maroc* 92: 1–372.

Cappetta, H. 2012. *Chondrichthyes - Mesozoic and Cenozoic Elasmobranchii: Teeth*. *In* Schultze, H.-P. (ed.) *Handbook of Paleoichthyology*. Verlag F. Pfeil, Munich, 512pp.

Cohen, K.M., Finney, S.C., Gibbard, P.L. & Fan, J.-X. 2013 (updated 2020). The ICS International Chronostratigraphic Chart. *Episodes* 36: 199-204.

Kocsis, L., Gheerbrant, E., Mouflih, M., Cappetta, H., Yans, J. and Amaghzaz, M. 2014. Comprehensive stable isotope investigation of marine biogenic apatite from the late Cretaceous–early Eocene phosphate series of Morocco. *Palaeogeography, Palaeoclimatology, Palaeoecology* 394: 74–88.

Noubhani, A. and Cappetta, H. 1997. Les Orectolobiformes, Carcharhiniformes et Myliobatiformes des bassins à phosphate du Maroc (Maastrichtien-Lutétien basal). Systématique, biostratigraphie, évolution et dynamique des faunes. *Palaeo Ichthyologica* 8: 1–327

Yans, J., Amaghzaz, M., Bouya, B., Cappetta, H., Iacumin, P., Kocsis, L., Mouflih, M., Selloum, O., Sen, S., Storme, J.-Y. and Gheerbrant, E. 2014. First carbon isotope chemostratigraphy of the Ouled Abdoun phosphate Basin, Morocco; implications for dating and evolution of earliest African placental mammals. *Gondwana Research* 25 (1): 257–269

Fossil taxon #3: †*Sphyrna* sp.

- **Node calibrated:** Crown node of family Sphyrnidae.
- **Node (see Fig. 4):** MRCA of Sphyrnidae (*Eusphyra blochii* + *Sphyrna* spp.).
- **Reference specimens (CR #1):** 40 specimens (including UM-PAD 123-126) reported by Cappetta & Case (2006), deposited in the collections of the University of Montpellier, France. The specimens are isolated teeth sampled from the Point 'A' Dam site, northwest of Andalusia city, Covington Co, Alabama.
- **Phylogenetic placement (CR #2 and CR #3):** Fossils unequivocally assigned to the family Sphyrnidae on the basis of a combination of several morphological (dental) characters (Cappetta & Case, 2006). A formal phylogenetic position of this species has not been established by morphological characters. The nominal species *Sphyrna guinoti* Adnet *et al.* 2020 has been very recently described from the Souar-Fortuna Formation in Tunisia, which is only slightly younger that Fossil taxon #3 and dated by radiometric K-Ar datings to middle Bartonian (38.7 ± 1.0 Ma to 40.7 ± 1.1 Ma) in Marivaux *et al.* (2014).
- **Minimum age:** 41.2 Myrs (Cohen et al. 2013).
- **Locality, stratigraphic and age information (CR #4 and CR #5):** These specimens were sampled from a fossil-rich coarse-grained glauconitic sand at the base of the Middle Lutetian basal Lisbon Fm. (contact with underlying Tallahatta Fm.). Biostratigraphic data indicate that this level belongs to the Upper NP14-NP 15 zones that roughly corresponds to the Middle Lutetian. We use a more conservative age estimate and consider the age of these fossils as Lutetian (41.2 – 47.8 Mya; Cohen *et al.* 2013). Therefore, we used the youngest age estimated for this stage for this calibration (41.2 Myrs).
- **Previous use(s) as calibration and Discussion:** Not used in any previous dating analysis.
- **Best practices score:** 4/5.

References

Adnet, S., Marivaux, L., Cappetta, H., Charruault, A.-L., Essid, E.M., Jiquel, S., Ammar, H.K., Marandat, B., Marzougui, W., Merzeraud, G., Temani, R., Vianey-Liaud, M. and Tabuce, R. 2020. Diversity and renewal of tropical elasmobranchs around the Middle Eocene Climatic Optimum (MECO) in North Africa: New data from the lagoonal deposits of Djebel el Kébar, Central Tunisia. *Palaeontologia Electronica*: 23(2):a38.

Cappetta, H. and Case, G.R. 2016. A selachian fauna from the Middle Eocene (Lutetian, Lisbon Formation) of Andalusia, Covington County, Alabama, USA. *Palaeontographica, Abt. A* 307 (1–6): 43–103.

Cohen, K.M., Finney, S.C., Gibbard, P.L. & Fan, J.-X. 2013 (updated 2020). The ICS International Chronostratigraphic Chart. *Episodes* 36: 199-204.

Marivaux, L., Essid, E.M., Marzougui, W., Ammar, H.K., Adnet, S., Marandat, B., Merzeraud, G., Ramdarshan, A., Tabuce, R., Vianey- Liaud, M. and Yans, J. 2014. A morphological intermediate between eosimiiform and simiiform primates from the late middle Eocene of Tunisia: Macroevolutionary and paleobiogeographic implications of early anthropoids. *American Journal of Physical Anthropology* 154 (3): 387–401.

Fossil taxon #4: †*Eypea leesi* Underwood & Ward, 2004

- **Node calibrated:** Crown node of order Carcharhiniformes.
- **Node (see Fig. 4):** MRCA of Carcharhiniformes (from *Poroderma africanum* to *Prionace glauca*).
- **Reference specimens (CR #1):** several hundreds of specimens (including holotype BMNH P. 66058 and paratypes P. 66059-65) reported by Underwood & Ward (2004), deposited in the Department of Palaeontology at The Natural History Museum, London (NHM). The type specimens are isolated teeth sampled at Watton Cliff, Dorset (UK).
- **Phylogenetic placement (CR #2 and CR #3):** The genus *Eypea* is unequivocally assigned to the order Carcharhiniformes on the basis of a combination of several morphological (dental) characters (Underwood & Ward, 2004). This species was originally tentatively assigned to the Recent carcharhiniform family Proscylliidae, but the characters provided are equivocal and this taxon would better be regarded as a Scyliorhinidae *sensu lato*. A formal phylogenetic position of this species has not been established by morphological characters. Two additional carcharhiniform (Scyliorhinidae *sensu lato*) species (*Palaeoscyllium tenuidens* Underwood & Ward, 2004, *Praeproscyllium oxoniensis* Underwood & Ward, 2004) were also reported by Underwood & Ward (2004), all sampled form British Bathonian formations.
- **Minimum age:** 166.1 Myrs (Cohen et al. 2013).
- **Locality, stratigraphic and age information (CR #4 and CR #5):** The holotype was sampled from a bioclastic limestone in the Forest Marble Formation of Bathonian age (Underwood & Ward, 2004) in the Great Oolite Group. The Bathonian stage spans the time between 168.3 and 166.1 Myrs ago (Cohen et al. 2013). Therefore, we used the youngest age estimated for this stage for this calibration (166.1 Myrs).
- **Previous use(s) as calibration and Discussion:** Not used in any previous dating analysis.
- **Best practices score:** 4/5.

References

Cohen, K.M., Finney, S.C., Gibbard, P.L. & Fan, J.-X. 2013 (updated 2020). The ICS International Chronostratigraphic Chart. *Episodes* 36: 199-204.

Underwood, C.J. and Ward, D.J. 2004. Neoselachian sharks and rays from the British Bathonian (Middle Jurassic). *Palaeontology* 47 (3): 447–501.

Fossil taxon #5: †*Isurolamna inflata* (Leriche, 1905)

- **Node calibrated:** Crown node of family Lamnidae.
- **Node (see Supplementary Data S11):** MRCA of Lamnidae (*Carcharodon carcharias* + *Lamna* spp. + *Isurus* spp.).
- **Reference specimens (CR #1):** several specimens (including P.6392-P.6396) reported by Baut & Genault (1995), deposited in the collections of the Royal Belgian Institute of Natural Sciences. The specimens are isolated teeth sampled from several localities (Rollot, Ressons-sur-Matz, Elincourt, and Antheuil-Portes) in the Oise and Somme departments, France.
- **Phylogenetic placement (CR #2 and CR #3):** The genus *Isurolamna* is unequivocally assigned to the family Lamnidae on the basis of a combination of several morphological (dental) characters (Cappetta, 1976). A formal phylogenetic position of this species has not been established by morphological characters.
- **Minimum age:** 56 Myrs (Cohen et al. 2013).
- **Locality, stratigraphic and age information (CR #4 and CR #5):** These specimens were sampled from a fossil-rich horizon (level 4) at the top of the informal 'Sables de Bracheux' formation, which are considered upper Thanetian in age (Baut & Genault, 1995). We use a more conservative age estimate and consider the age of these fossils as Thanetian (56–59.2 Mya; Cohen *et al.* 2013). Therefore, we used the youngest age estimated for this stage for this calibration (56 Myrs). The genus *Isurolamna* was also reported from the Thanetian and possibly Danian of Morocco (Noubhani & Cappetta, 1997) but these specimens were not illustrated.
- **Previous use(s) as calibration and Discussion:** Not used in any previous dating analysis.
- **Best practices score:** 4/5.

References

Cappetta, H. 1976. Sélaciens nouveaux du London clay de l'Essex (Yprésien du Bassin de Londres). Geobios 9 (5): 551–575.

Baut, J.-P. and Genault, B. 1995. Contribution à l'étude des élasmobranches du Thanétien (Paléocène) du Bassin de Paris. 1. Découverte d'une faune d'élasmobranches dans la partie supérieure des Sables de Bracheux (Thanétien, Paléocène) des régions de Compiègne (Oise) et de Montdidier (Somme). *In*: Herman, J. and Van Waes, H. (eds.), *Elasmobranches et Stratigraphie*, 185–259. Professional Paper of the Belgian Geological Survey, Brussels.

Cohen, K.M., Finney, S.C., Gibbard, P.L. & Fan, J.-X. 2013 (updated 2020). The ICS International Chronostratigraphic Chart. *Episodes* 36: 199-204.

Leriche, M. 1905. Les poissons tertiaires de la Belgique. II. Les poissons éocènes. *Mémoires du Musée royal d'Histoire naturelle de Belgique* 3 (11): 49–228.

Noubhani, A. and Cappetta, H. 1997. Les Orectolobiformes, Carcharhiniformes et Myliobatiformes des bassins à phosphate du Maroc (Maastrichtien-Lutétien basal). Systématique, biostratigraphie, évolution et dynamique des faunes. *Palaeo Ichthyologica* 8: 1–327

Fossil taxon #6: †*Protolamna* sp.

- **Node calibrated:** Crown node of order Lamniformes.
- **Node (see Supplementary Data S11):** MRCA of Lamniformes (from *Mitsukurina owstoni* to *Carcharodon carcharias*).
- **Reference specimens (CR #1):** nine specimens (including ZPAL P.10⁄8-10) reported by Rees (2005), housed in the Institute of Paleobiology of the Polish Academy of Sciences, Warszawa (ZPAL). The specimens are isolated teeth sampled from the village of Wawał in central Poland, some 110 km south-west of Warsaw.
- **Phylogenetic placement (CR #2 and CR #3):** The genus *Protolamna* is unequivocally assigned to the order Lamniformes on the basis of a combination of several morphological (dental) characters (Cappetta, 1980, 2012). A formal phylogenetic position of this species has not been established by morphological characters. The Late Jurassic species †*Palaeocarcharias stromeri*, previously regarded as a potential lamniform *incertae sedis* is not used here as it is now placed in a separated order Landemaine et al. (2018).
- **Minimum age:** 132.6 Myrs (Cohen et al. 2013).
- **Locality, stratigraphic and age information (CR #4 and CR #5):** These specimens were sampled from a fossil-rich horizon (level W1) in the Seynoceras verrucosum Zone indicative of an Upper Valangnian age (Rees, 2005). We use a more conservative age estimate and consider the age of these fossils as Valanginian (139.8-132.6 Mya; Cohen *et al.* 2013). Therefore, we used the youngest age estimated for this stage for this calibration (132.6 Myrs).
- **Previous use(s) as calibration and Discussion:** Not used in any previous dating analysis.
- **Best practices score:** 4/5.

References

Cappetta, H. 1980. Modification du statut générique de quelques espèces de sélaciens crétacés et tertiaires. *Palaeovertebrata* 10 (1): 29–42.

Cappetta, H. 2012. *Chondrichthyes - Mesozoic and Cenozoic Elasmobranchii: Teeth*. *In* Schultze, H.-P. (ed.) *Handbook of Paleoichthyology*. Verlag F. Pfeil, Munich, 512pp.

Cohen, K.M., Finney, S.C., Gibbard, P.L. & Fan, J.-X. 2013 (updated 2020). The ICS International Chronostratigraphic Chart. *Episodes* 36: 199-204.

Landemaine, O., Thies, D. and Waschkewitz, J. 2018. The Late Jurassic shark *Palaeocarcharias* (Elasmobranchii, Selachimorpha) – functional morphology of teeth, dermal cephalic lobes and phylogenetic position. *Palaeontographica*, *Abt. A* 312 (5–6): 103–165.

Rees, J. 2005. Neoselachian shark and ray teeth from the Valanginian, Lower Cretaceous, of Wawal, central Poland. *Palaeontology* 48 (2): 209–221.

Fossil taxon #7: †*Reifia minuta* Duffin, 1980

- **Node calibrated:** Root of the tree.
- **Node (see Supplementary Data S11):** MRCA of Carcharhiniformes + Lamniformes (from *Mitsukurina owstoni* to *Prionace glauca*).
- **Reference specimens (CR #1):** five specimens (including holotype 50.200 and paratypes 50.201-204) reported by Duffin (1980), deposited in the Seilacher collection of the Paleontology Department of the Staatliches Museum für Naturkunde, Ludwigsburg, W Germany. The type specimens are isolated teeth sampled from the Eisbach Valley, near Gaildorf (SW Germany).
- **Phylogenetic placement (CR #2 and CR #3):** Teeth of this taxon encompass a set of characters of which some are considered apomorphies of Orectolobiformes and others of Carcharhiniformes (Cappetta, 2012; Duffin, 1980). This taxon is considered as a Galeomorphii *incertae sedis*.
- **Minimum age:** 208.5 Myrs (Cohen et al. 2013).
- **Locality, stratigraphic and age information (CR #4 and CR #5):** The type specimens were sampled from the marly horizon in the Dunkel Mergel, Lower Norian. The Norian stage spans the time between 227 and 208.5 Myrs ago (Cohen et al. 2013). Therefore, we used the youngest age estimated for this stage for this calibration (208.5 Myrs).
- **Previous use(s) as calibration and Discussion:** Not used in any previous dating analysis.
- **Best practices score:** 4/5.

References

Cappetta, H. 2012. *Chondrichthyes - Mesozoic and Cenozoic Elasmobranchii: Teeth*. *In* Schultze, H.-P. (ed.) *Handbook of Paleoichthyology*. Verlag F. Pfeil, Munich, 512pp.

Cohen, K.M., Finney, S.C., Gibbard, P.L. & Fan, J.-X. 2013 (updated 2020). The ICS International Chronostratigraphic Chart. *Episodes* 36: 199-204.

Duffin, C.J. 1980. A new euselachian shark from the Upper Triassic of Germany. *Neues Jahrbuch für Geologie und Paläontologie, Monatshefte* 1980 (1): 1–16.
