# Supplementary material for: Combining palaeontological and neontological data shows a delayed diversification burst of carcharhiniform sharks likely mediated by environmental change
Source: Sci Rep. 2022 Dec 19;12:21906. doi: 10.1038/s41598-022-26010-7 (PMC9763247; doi:10.1038/s41598-022-26010-7)
Supplement: Supplementary file 5 — Supplementary Information 5. [file 41598_2022_26010_MOESM5_ESM.zip › Supplementary Data S5 - Ts comparaison 35 spp/Supplementary Data S5 - Ts comparaison 35 spp/Legend.docx]

**Supplementary Data S5.** Comparison of times of speciation estimated with PyRate and dating analyses for the 35 extant species represented in the fossil record.
