# Supplementary material for: Combining palaeontological and neontological data shows a delayed diversification burst of carcharhiniform sharks likely mediated by environmental change
Source: Sci Rep. 2022 Dec 19;12:21906. doi: 10.1038/s41598-022-26010-7 (PMC9763247; doi:10.1038/s41598-022-26010-7)

Supplementary Data S11. Time-calibrated phylogenies of Carcharhiniformes. The chronograms present the results from the BEAST analyses that simultaneously estimated the topology and divergence times for the Carcharhiniformes and Lamniformes. We performed four analyses to take into account the effects of the number of molecular clocks and of the maximum age at the tree root (see Methods). We compared dating analyses made with four molecular clocks versus dating analyses made with seven molecular clocks. We also evaluated the effect of maximum age at the root when set to 208.5 Ma or to 251.9 Ma.

**210 sharks, 7 fossils calibrations, 4 clocks 208 Ma max age**

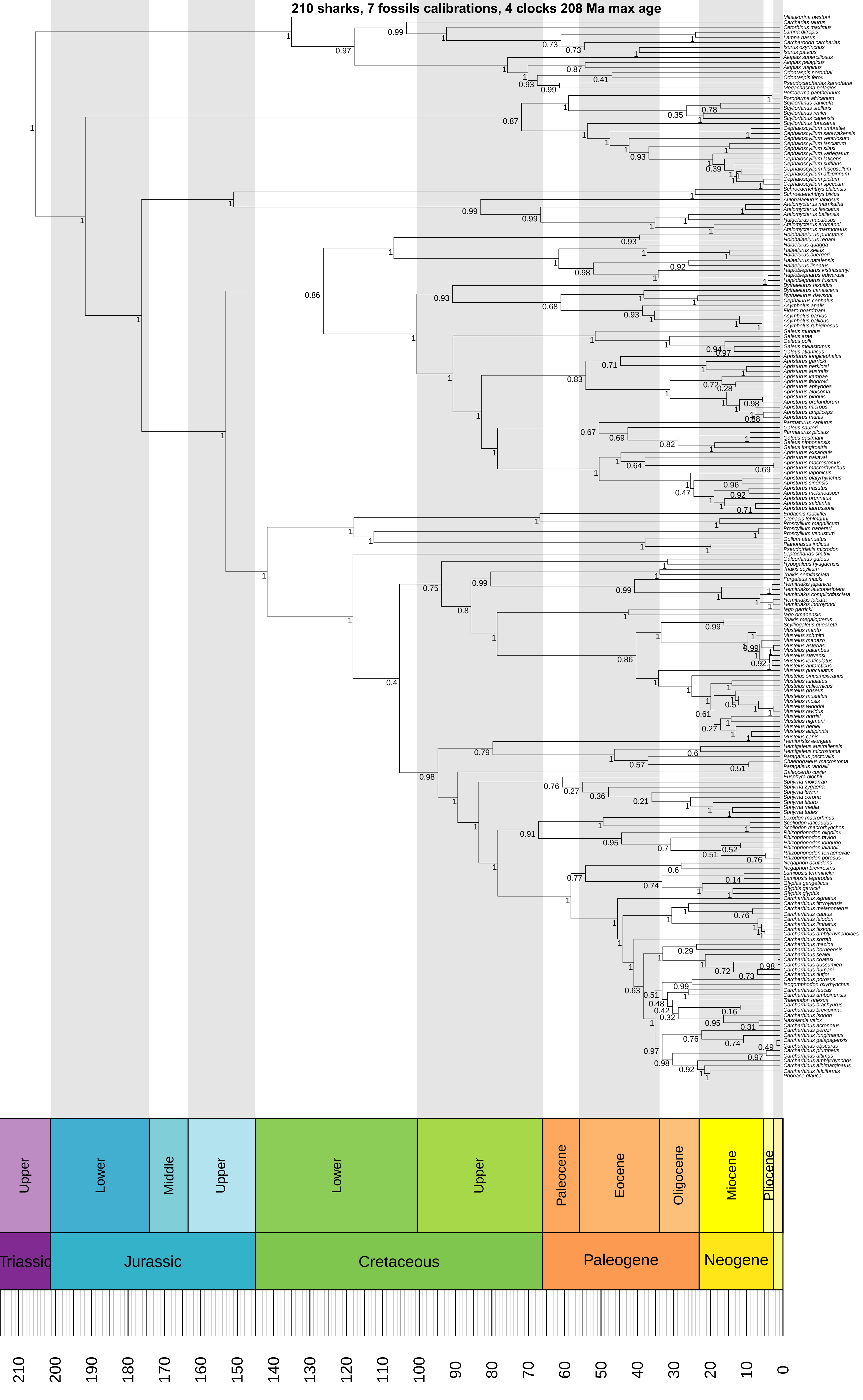

## 210 sharks, 7 fossils calibrations, 4 clocks 251 Ma max age

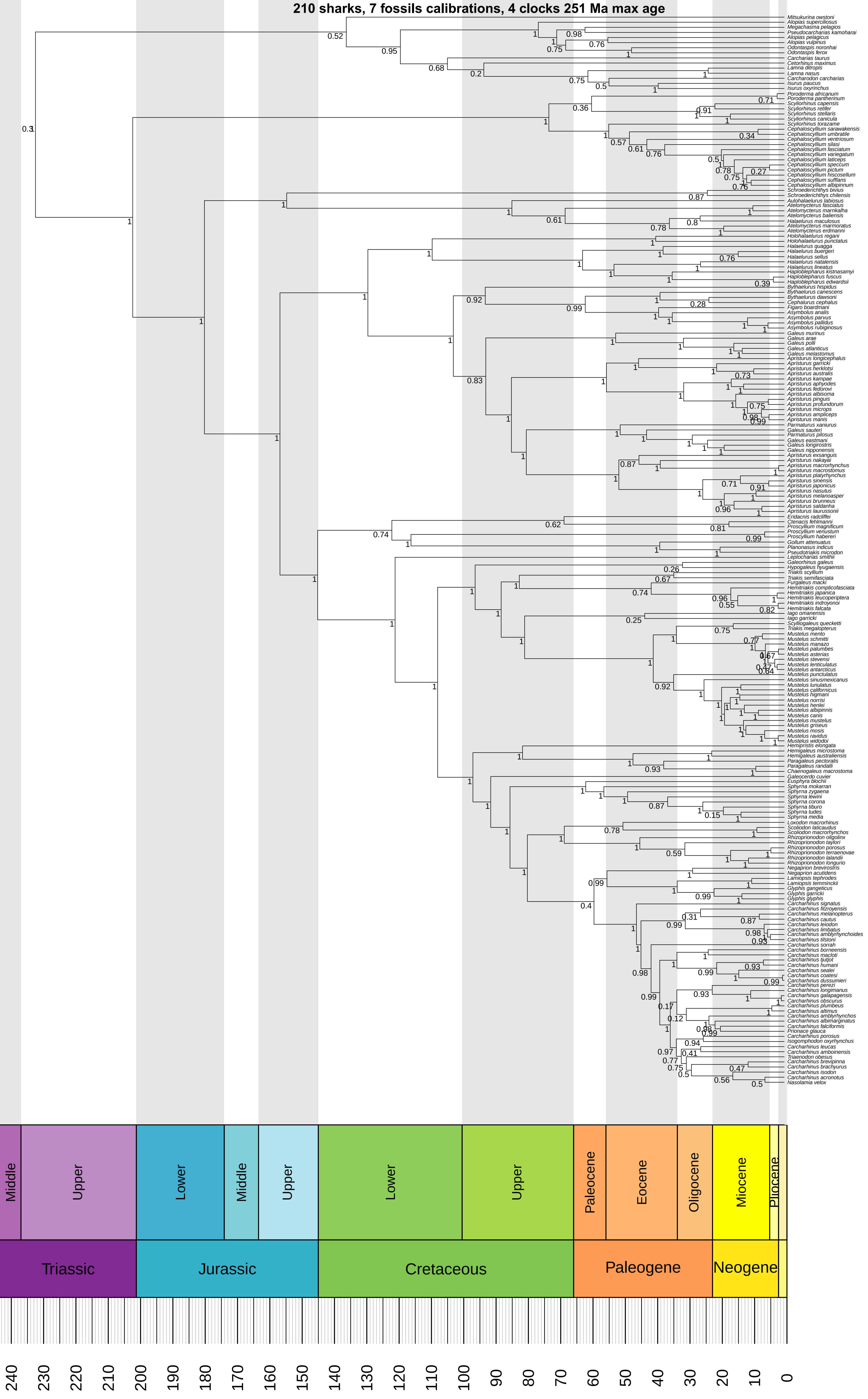

## 210 sharks, 7 fossils calibrations, 7 clocks 208 Ma max age

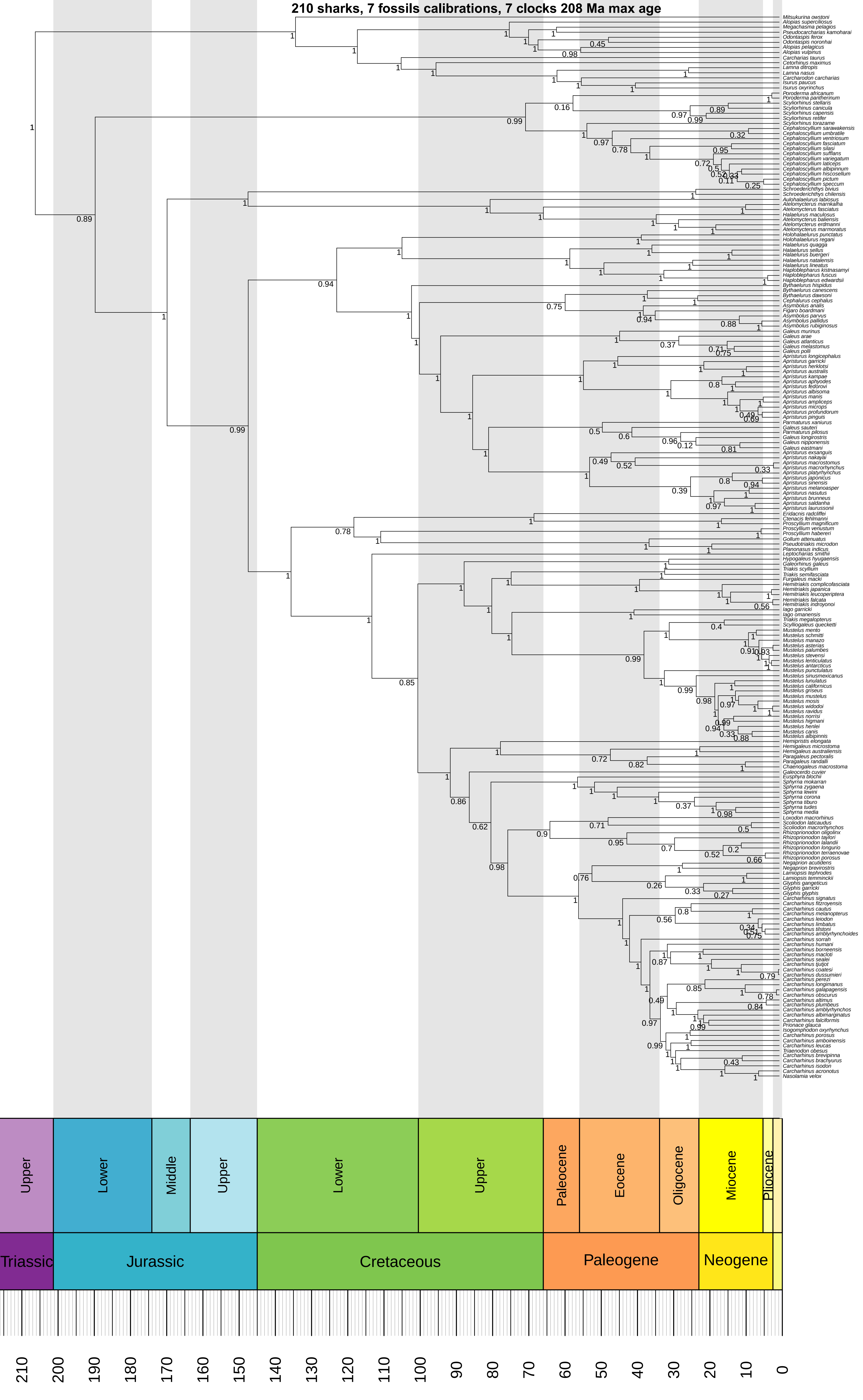

## 210 sharks, 7 fossils calibrations, 7 clocks 251 Ma max age

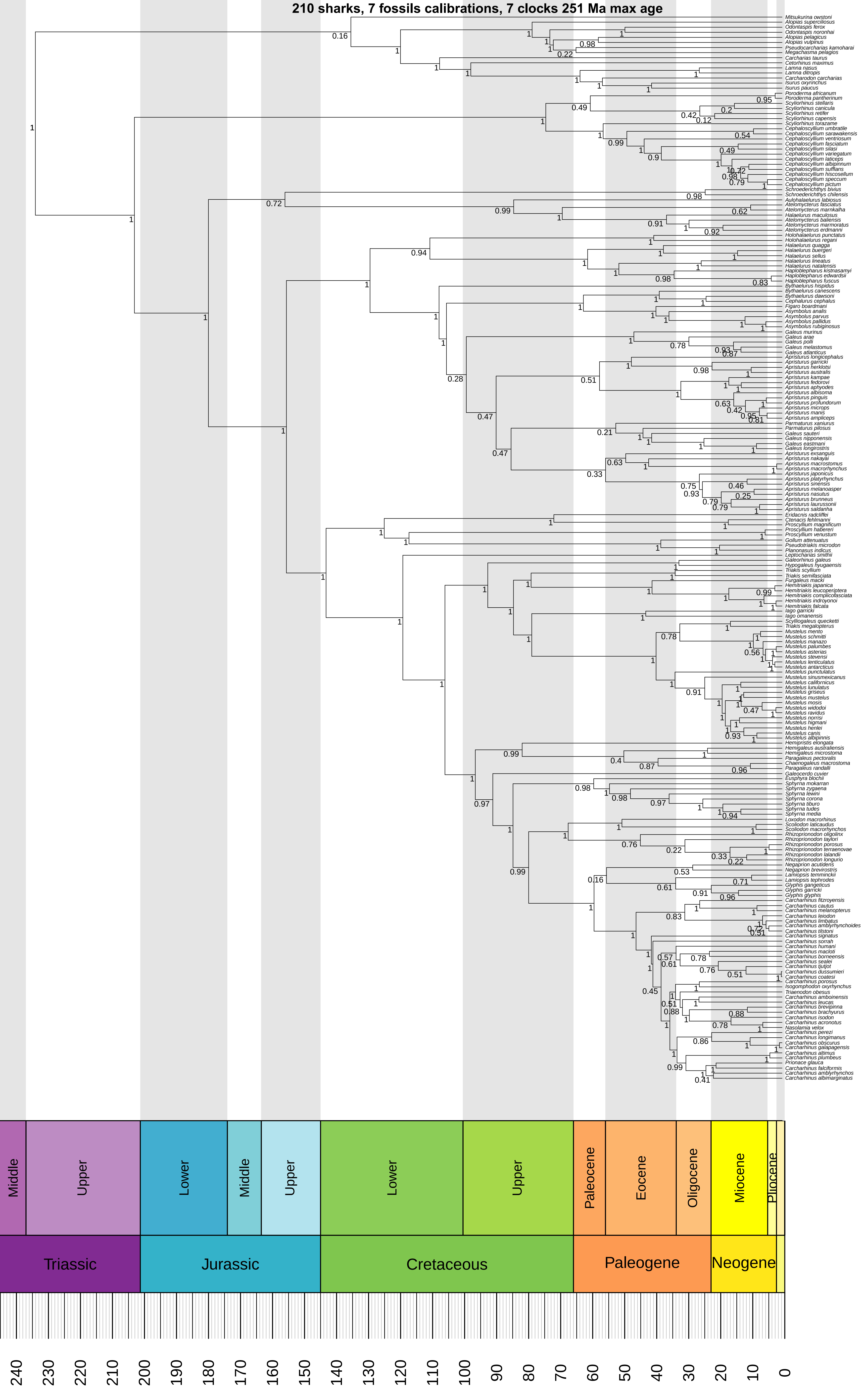

Supplement: Supplementary file 11 — Supplementary Information 11. [file 41598_2022_26010_MOESM11_ESM.pdf]
