# Supplementary material for: Combining palaeontological and neontological data shows a delayed diversification burst of carcharhiniform sharks likely mediated by environmental change
Source: Sci Rep. 2022 Dec 19;12:21906. doi: 10.1038/s41598-022-26010-7 (PMC9763247; doi:10.1038/s41598-022-26010-7)
Supplement: Supplementary file 12 — Supplementary Information 12. [file 41598_2022_26010_MOESM12_ESM.pdf]

Supplementary Data S12. Posterior estimates and corresponding 95% HPD of the preservation rate (*q*).

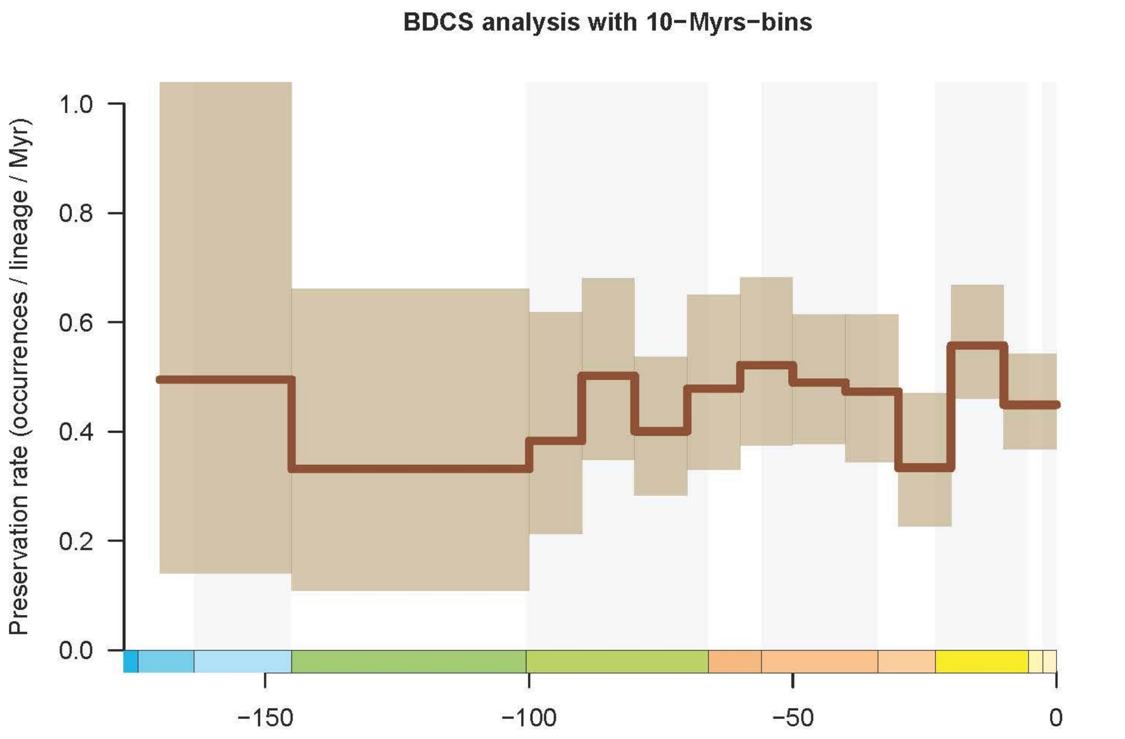

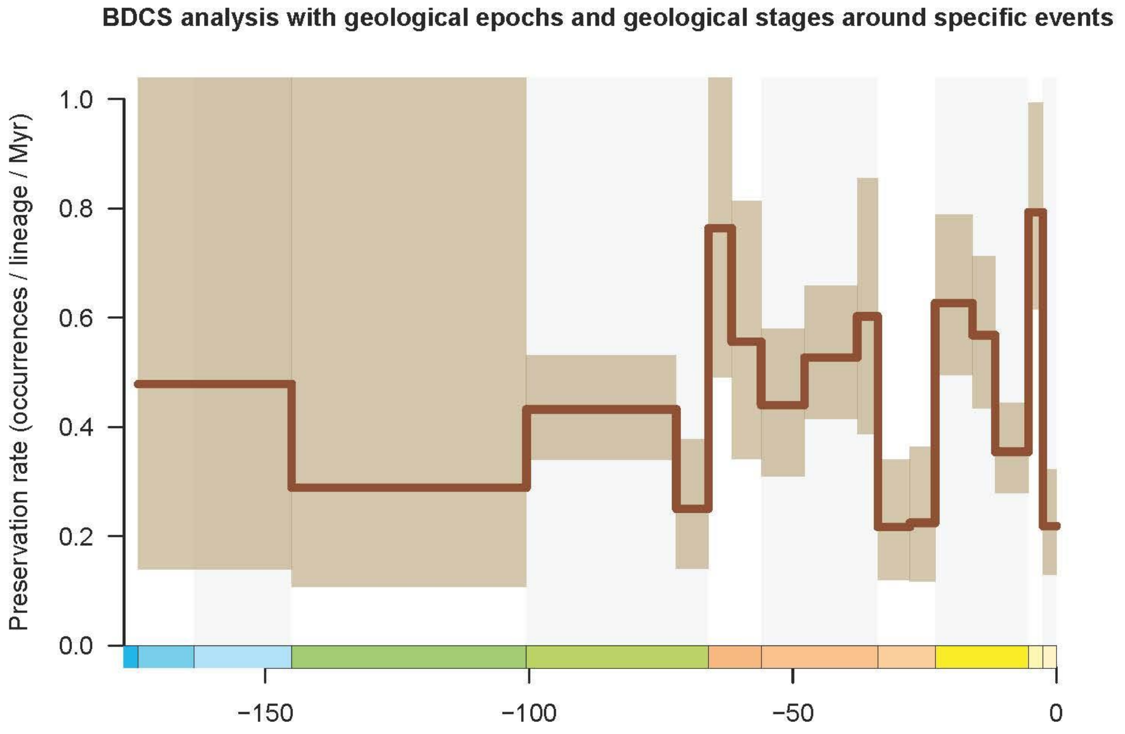

Figure S1. Temporal variations of the preservation rates (*q*) estimated under the BDCS-Fossils analyses performed over 10 replicated datasets of the carcharhiniform fossil record using 10 Myrs time bins (upper panel) and times bins corresponding to geological epochs and geological stages around specific events (lower panel). Solid lines indicate mean posterior rates and shaded areas show 95% HPD.
