# Supplementary material for: Combining palaeontological and neontological data shows a delayed diversification burst of carcharhiniform sharks likely mediated by environmental change
Source: Sci Rep. 2022 Dec 19;12:21906. doi: 10.1038/s41598-022-26010-7 (PMC9763247; doi:10.1038/s41598-022-26010-7)
Supplement: Supplementary file 14 — Supplementary Information 14. [file 41598_2022_26010_MOESM14_ESM.pdf]

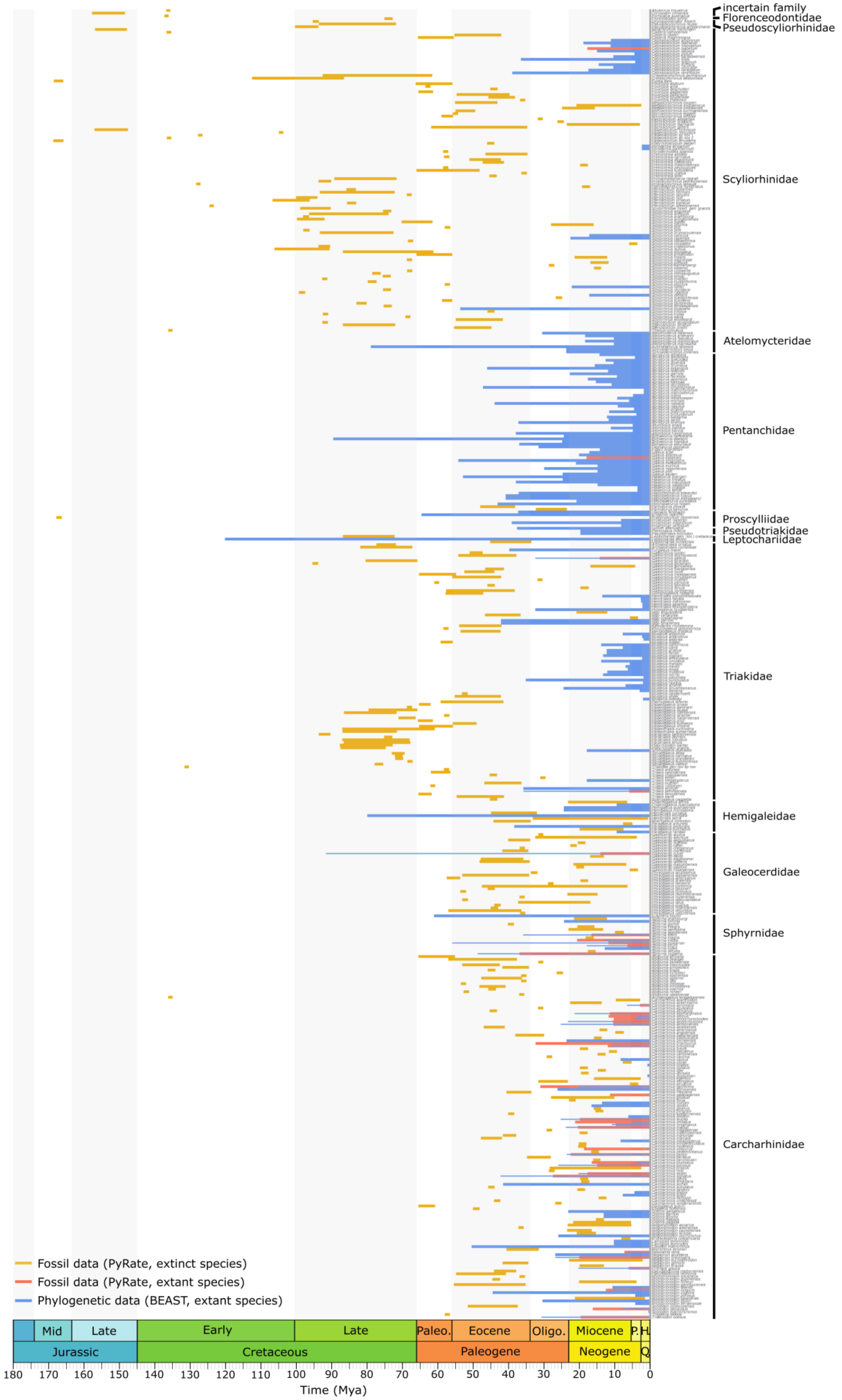

Supplementary Data S14. Estimates of species lifespan from the fossil record and the molecular phylogeny for the Carcharhiniformes. Species lifespan is determined by the times of speciation (*Ts*) and times of extinction (*Te*), as inferred with the fossil record for extinct species (yellow) and extant species having fossil occurrences (orange), and those inferred with the dated phylogeny for extant species only (blue). Summing up the number of species per million-year bins allows recovering the diversity dynamics of the clade through time.
