# Supplementary material for: Combining palaeontological and neontological data shows a delayed diversification burst of carcharhiniform sharks likely mediated by environmental change
Source: Sci Rep. 2022 Dec 19;12:21906. doi: 10.1038/s41598-022-26010-7 (PMC9763247; doi:10.1038/s41598-022-26010-7)
Supplement: Supplementary file 15 — Supplementary Information 15. [file 41598_2022_26010_MOESM15_ESM.pdf]

Supplementary Data S15. BDCS-Combined

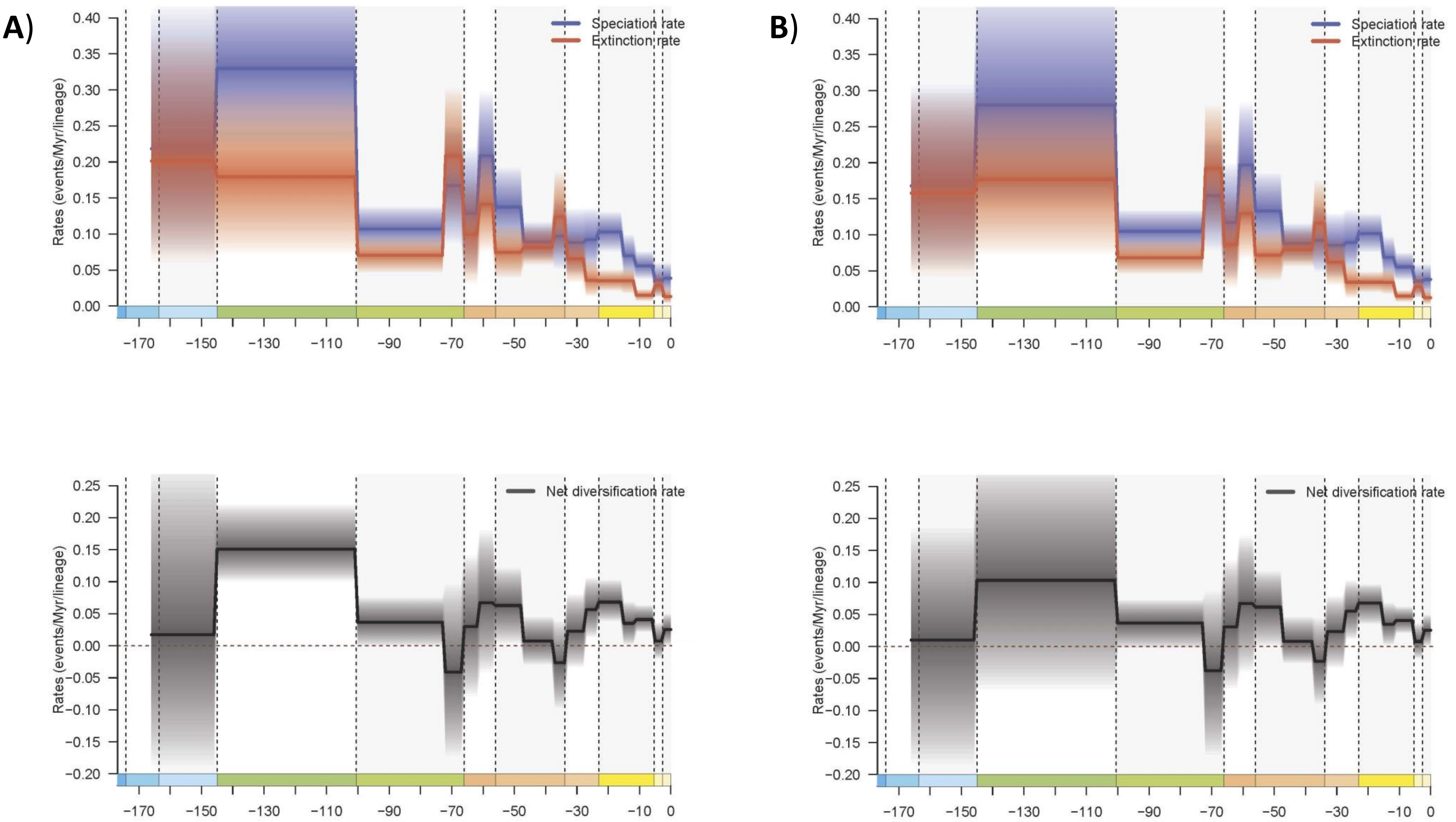

Figure S1. Diversification dynamics of Carcharhiniformes estimated with the combined dataset (BDCS-Combined) and geological epochs (and stages in the Cenozoic). Inferences of speciation (blue) and extinction (red) rates with analyses at the species level under the birth–death model with constrained shifts. The net diversification rates (black) are the difference between speciation and extinction rates (rates below 0 indicate declining diversity). Solid lines indicate mean posterior rates and the shaded areas show 95% HPD. The results show negative net diversification rates that lead to a diversity decline for the group at the Cretaceous-Paleogene and Eocene-Oligocene boundaries, and also in the mid-Miocene and the last 5 million years. **A)** Estimates using the -*N* parameter; **B)** estimates without the -*N* parameter.

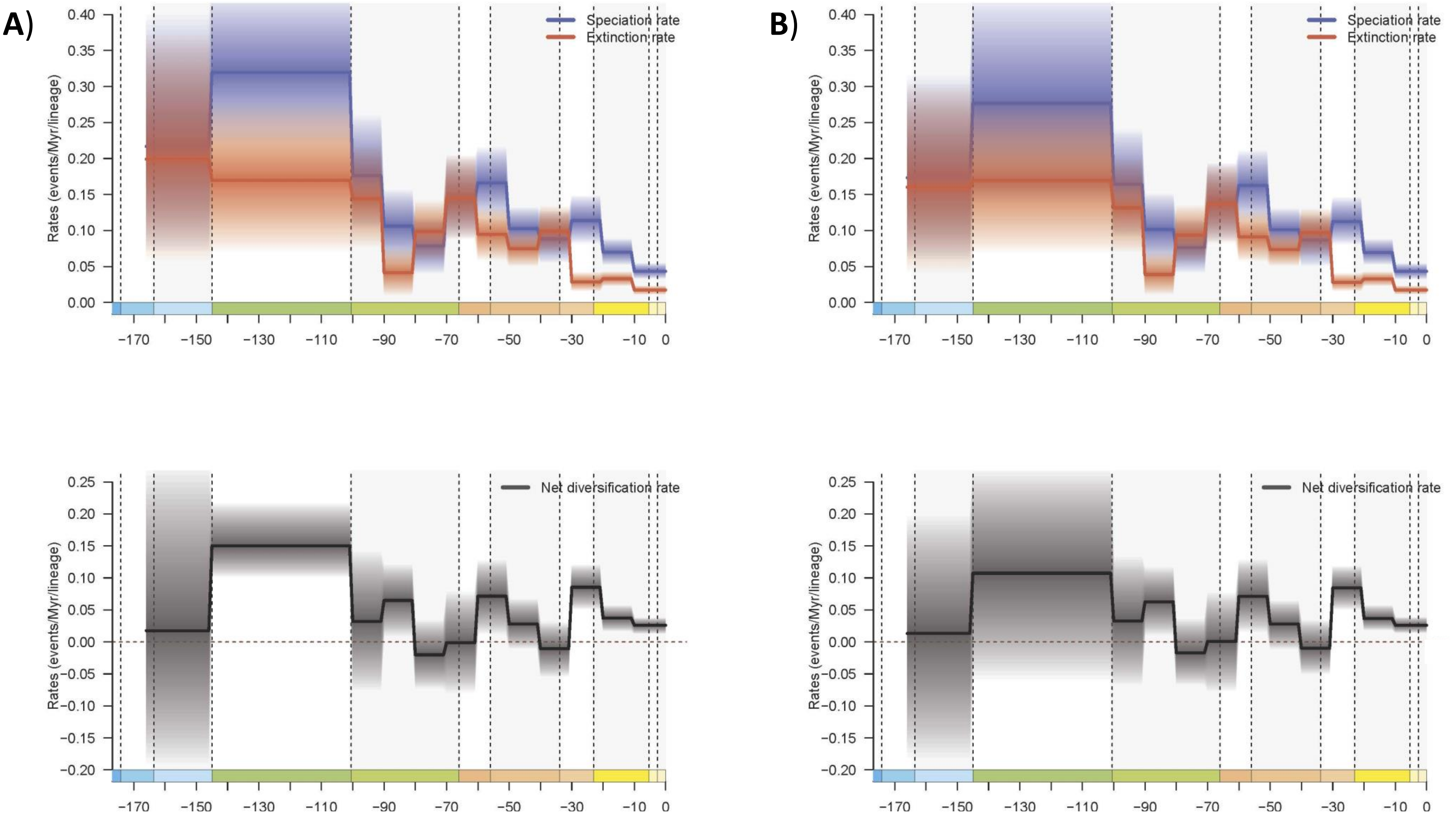

Figure S2. Diversification dynamics of Carcharhiniformes estimated with the combined dataset (BDCS-Combined) with 10 Myrs time bins. Inferences of speciation (blue) and extinction (red) rates with analyses at the species level under the birth–death model with constrained shifts. The net diversification rates (black) are the difference between speciation and extinction rates (rates below 0 indicate declining diversity). Solid lines indicate mean posterior rates and the shaded areas show 95% HPD. The results show negative net diversification rates that lead to a diversity decline for the group at the Cretaceous-Paleogene and Eocene-Oligocene boundaries, and also in the mid-Miocene and the last 5 million years. **A)** Estimates using the -*N* parameter; **B)** estimates without the -*N* parameter.
