# Supplementary material for: Combining palaeontological and neontological data shows a delayed diversification burst of carcharhiniform sharks likely mediated by environmental change
Source: Sci Rep. 2022 Dec 19;12:21906. doi: 10.1038/s41598-022-26010-7 (PMC9763247; doi:10.1038/s41598-022-26010-7)
Supplement: Supplementary file 16 — Supplementary Information 16. [file 41598_2022_26010_MOESM16_ESM.pdf]

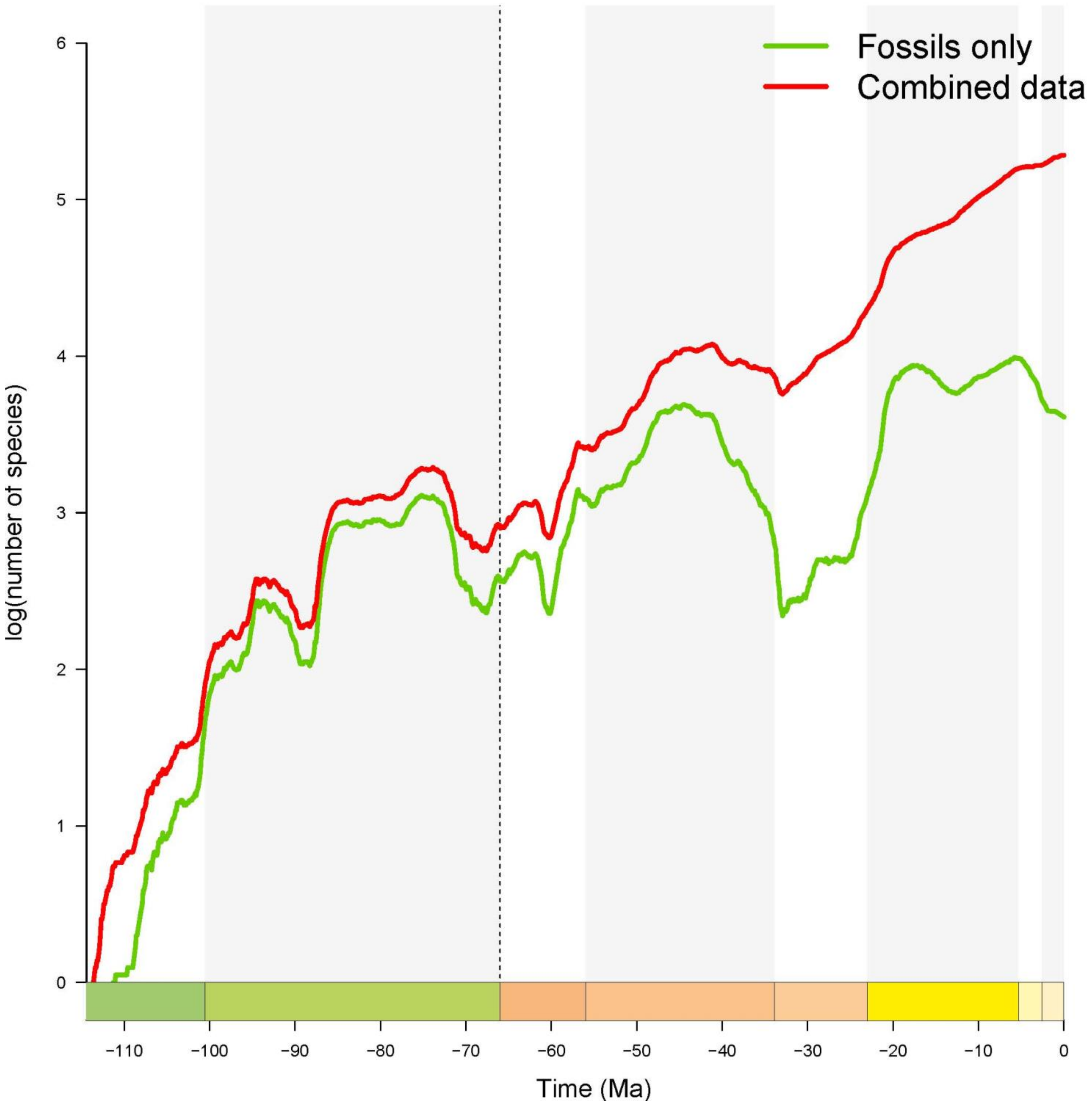

Supplementary Data S16. Comparison of log-transformed diversity (number of species) through time as estimated with fossils only and combined data (fossil and phylogenetic data). The number of species through time is plotted from 110 Ma onward since they do not differ before.
