# Supplementary material for: Combining palaeontological and neontological data shows a delayed diversification burst of carcharhiniform sharks likely mediated by environmental change
Source: Sci Rep. 2022 Dec 19;12:21906. doi: 10.1038/s41598-022-26010-7 (PMC9763247; doi:10.1038/s41598-022-26010-7)
Supplement: Supplementary file 17 — Supplementary Information 17. [file 41598_2022_26010_MOESM17_ESM.pdf]

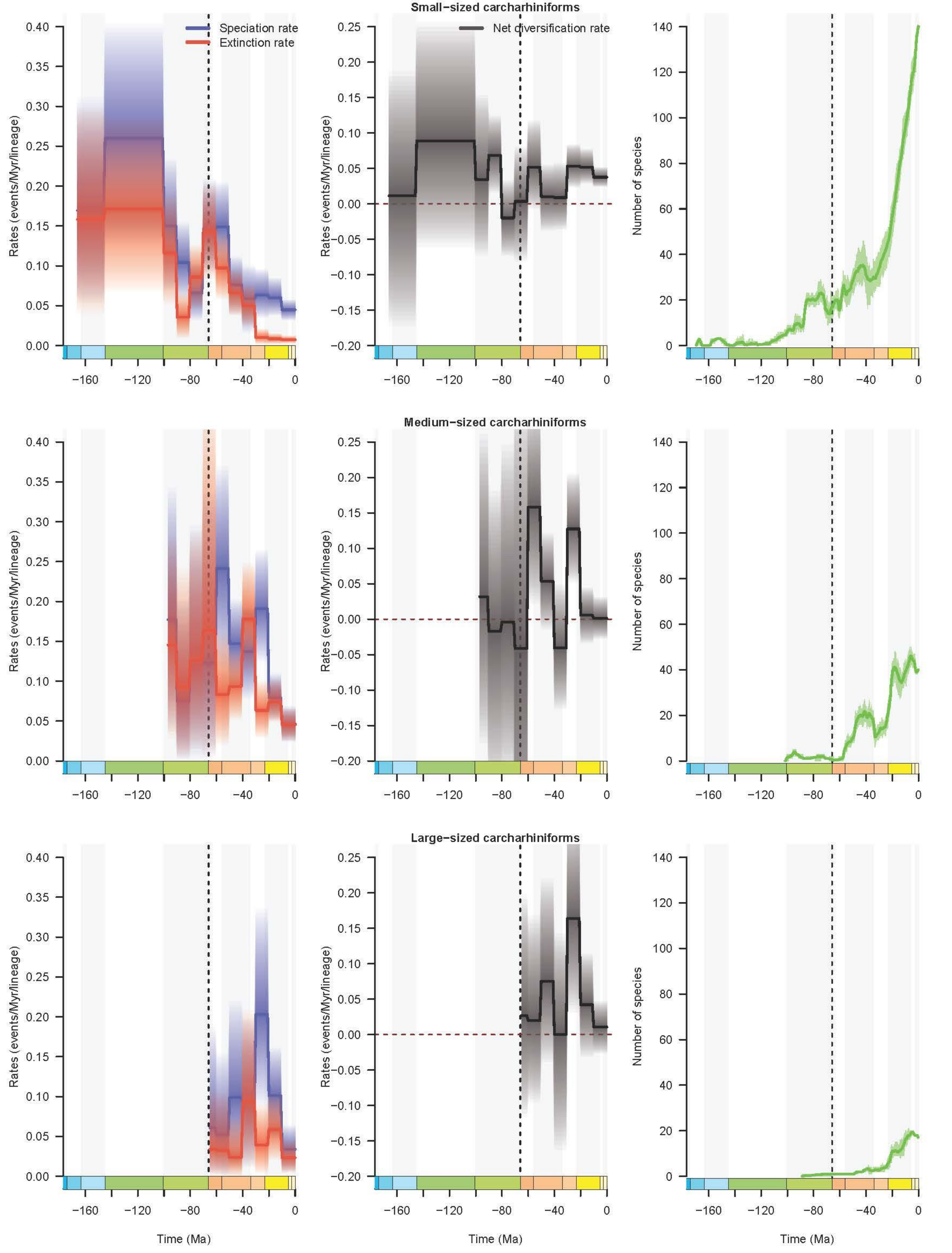

Supplementary Data S17. Diversification dynamics of three carcharhiniform classes based on tooth size (proxy for ecological niches) estimated with the fossil and phylogenetic dataset. Inferences of speciation (blue) and extinction (red) rates with analyses at the species level under the birth–death model with constrained shifts every 10 million years. The net diversification rates (black) are the difference between speciation and extinction rates (rates below 0 indicate declining diversity). Solid lines indicate mean posterior rates and the shaded areas show 95% HPD. The size-class diversity trajectories (green) incorporate uncertainties around the age of the fossil occurrences
