# Supplementary material for: Combining palaeontological and neontological data shows a delayed diversification burst of carcharhiniform sharks likely mediated by environmental change
Source: Sci Rep. 2022 Dec 19;12:21906. doi: 10.1038/s41598-022-26010-7 (PMC9763247; doi:10.1038/s41598-022-26010-7)
Supplement: Supplementary file 20 — Supplementary Information 20. [file 41598_2022_26010_MOESM20_ESM.pdf]

Supplementary Data S20. Compatibility between fossil- and phylogeny-based diversification rates.

Method

We jointly analyzed the fossil and phylogenetic data with the birth-death chronospecies (BDC) model implemented in PyRate 3 (Silvestro et al. 2018). The BDC model allows estimating whether alternative speciation modes described for the fossil record, that is, budding, bifurcation, or anagenesis (Silvestro et al. 2018), are responsible for driving incongruences between fossil and phylogenetic estimates. We compared an (1) *Equal rates model* where speciation and extinction parameters estimated with phylogenetic data (λ and μ, respectively) are the same to those estimated with stratigraphic data (λ^* and μ^*), that is, $\lambda^*=\lambda$, $\mu^*=\mu$; (2) a *Compatible model* where parameters differ, but the differences could be explained by differences in speciation mode. In this model, λ^*, λ, μ, and μ^* are constrained such that $\lambda^*-\lambda=\mu^*-\mu$ (i.e. equal net diversification rates) and $\lambda^*\geq\lambda$; and (3) an *Incompatible model,* where parameters λ, λ^*, μ, and μ^* are allowed to take any value and thus differences in λ and λ^*, as well as μ and μ^*, cannot be explained by differences in speciation mode.

We first estimated λ, μ, λ^*, and μ^* simultaneously for the species-level phylogeny and the corresponding fossil data using maximum-likelihood optimization while assuming constant diversification rates through time. To assess support for the BDC model, we applied a likelihood ratio test (Silvestro et al. 2018), comparing which of the equal, compatible, or incompatible rate models are supported by our data. Because the fossil record of carcharhiniforms dates back to the Middle Jurassic and shows substantial temporal heterogeneity in both speciation and extinction rates (see Results), we secondly implemented a Bayesian skyline model with rate shifts as defined with the 10-million-years (Myrs) time bins (resulting in 16 time bins). For each BDC model, we ran 10 million MCMC iterations to obtain joint posterior distributions of the

stratigraphic and phylogenetic rates. We set the fraction of sampled extant species in the phylogeny over the total extant species (-sampling option set to 0.687). We used the joint posterior samples of λ^*, μ^*, λ, and μ obtained under the incompatible rates model to verify the conditions predicted by the compatible BDC model (i.e. $\lambda^*-\lambda=\mu^*-\mu$ and $\lambda^*\geq\lambda$) and assess the support for each model as suggested in previous studies (Silvestro et al. 2018).

If the *Compatible rates* or *Equal rates* model fits best the two types of data in the BDC analyses, there are no major incongruences between fossil and phylogenetic rate estimates. In these cases, we argue that fossil and phylogenetic data can be combined in a single framework to provide more comprehensive estimates of the diversification pattern. On the contrary, if the *Incompatible rates* model best fits the data, this suggests that there are discrepancies (e.g. taxonomic incongruences) between modern and fossil data that lead to conflicts in rate estimates, thus rendering the combination of fossil and phylogenetic data impossible.

Results

Applying the BDC model with constant rates to both the carcharhiniform fossil and phylogenetic data at the species-level, we meet the expectations of the compatible rates model with $\lambda^*-\lambda=\mu^*-\mu$ and $\lambda^*\geq\lambda$ (Figure S1; *P*=0.11). Although fossil-based and phylogeny-based estimates of speciation and extinction rates showed large discrepancies, these can be attributed to the occurrence of bifurcating and anagenetic speciation events, without the need to invoke any potential biases in the data. However, this shark order is characterized by an ancient evolutionary history and exhibits a substantial amount of temporal variation in both speciation and extinction rates (see below), which can result in spurious support of a constant-rate BDC model. We re-analyzed the datasets using a time-variable implementation of the BDC model, which allows for rate variation across different intervals. Relaxing the assumption of

constant rates resulted in strong support for the BDC model within 13 of 16 time bins (P>0.1) and moderate support for the three remaining time bins (P>0.05) used in the analysis (Figure S2). All time bins comply with rate congruence between molecular and fossil data. Both fossil and phylogenetic data supported significant rate heterogeneity through time (Figure S3). Finally, we find evidence that budding significantly exceeds anagenetic origination throughout most of the Cenozoic diversification history of the group. The compatibility between fossil and molecular diversification rates allows us to combine the two datasets to assess the evolutionary history of Carcharhiniformes.

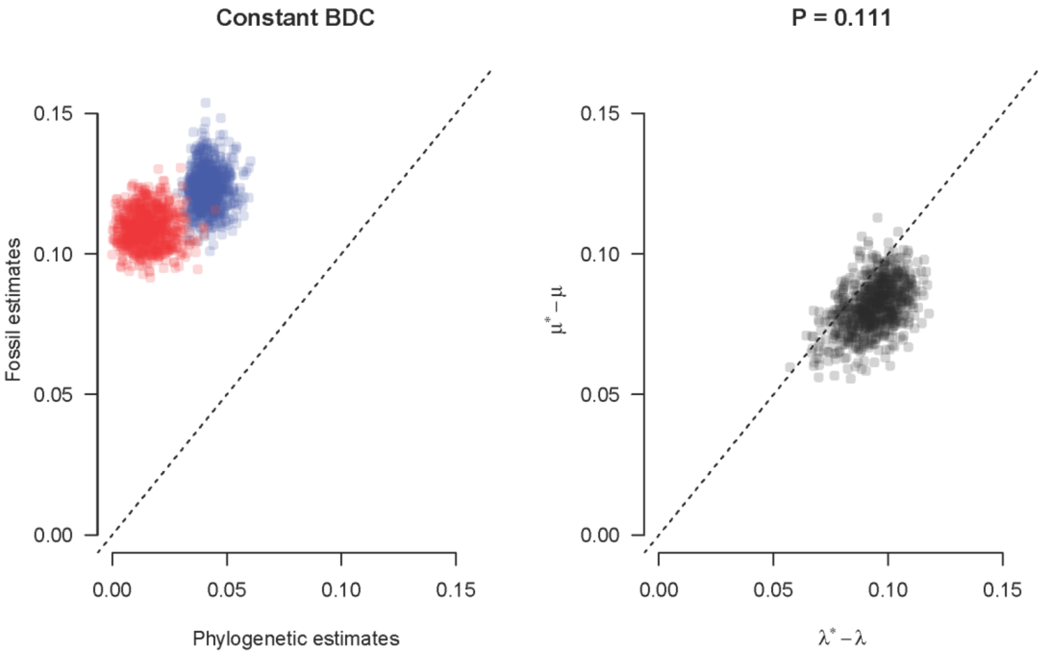

Figure S1. Results of the Bayesian Birth-Death Chronospecies (BDC) applied to fossil and phylogenetic data of Carcharhiniformes. Posterior distributions of speciation rates (in blue) and extinction rates (in red) jointly inferred from the two data types are plotted against one another, and posterior distributions of $\lambda^*-\lambda=\mu^*-\mu$ are shown in black. The results shown here are based on the assumption of constant rates through time (see Supplementary data 10 and 11 for the time-variable BDC model). Although the analyses were run assuming independent rates (λ, μ, λ^*, μ^*), their joint posterior distributions were used to assess which model (equal rates, compatible rates, or incompatible rates) best fit the data. Here, the compatible rates model is supported with fossil-based and phylogeny-based speciation and extinction rates being different, but compatible with the expectations of the BDC model. Accounting for rate variation through time, we also find support for the compatible rates model in most time bins (see Figures S2-3 below).

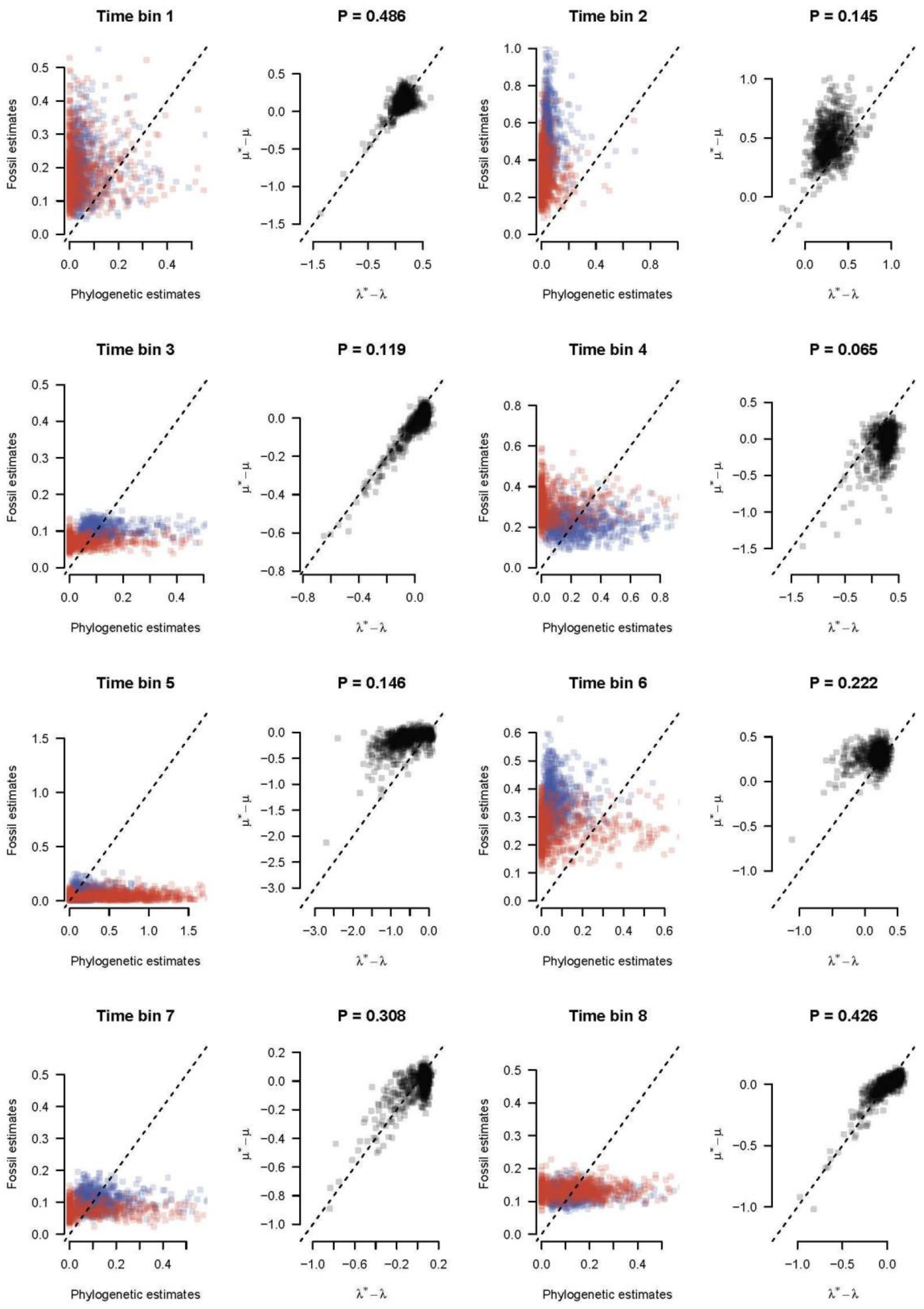
Time bin 1
P = 0.486
Time bin 2
P = 0.145
Time bin 3
P = 0.119
Time bin 4
P = 0.065
Time bin 5
P = 0.146
Time bin 6
P = 0.222
Time bin 7
P = 0.308
Time bin 8
P = 0.426
Fossil estimates
Phylogenetic estimates
$\mu^* - \mu$
$\lambda^* - \lambda$

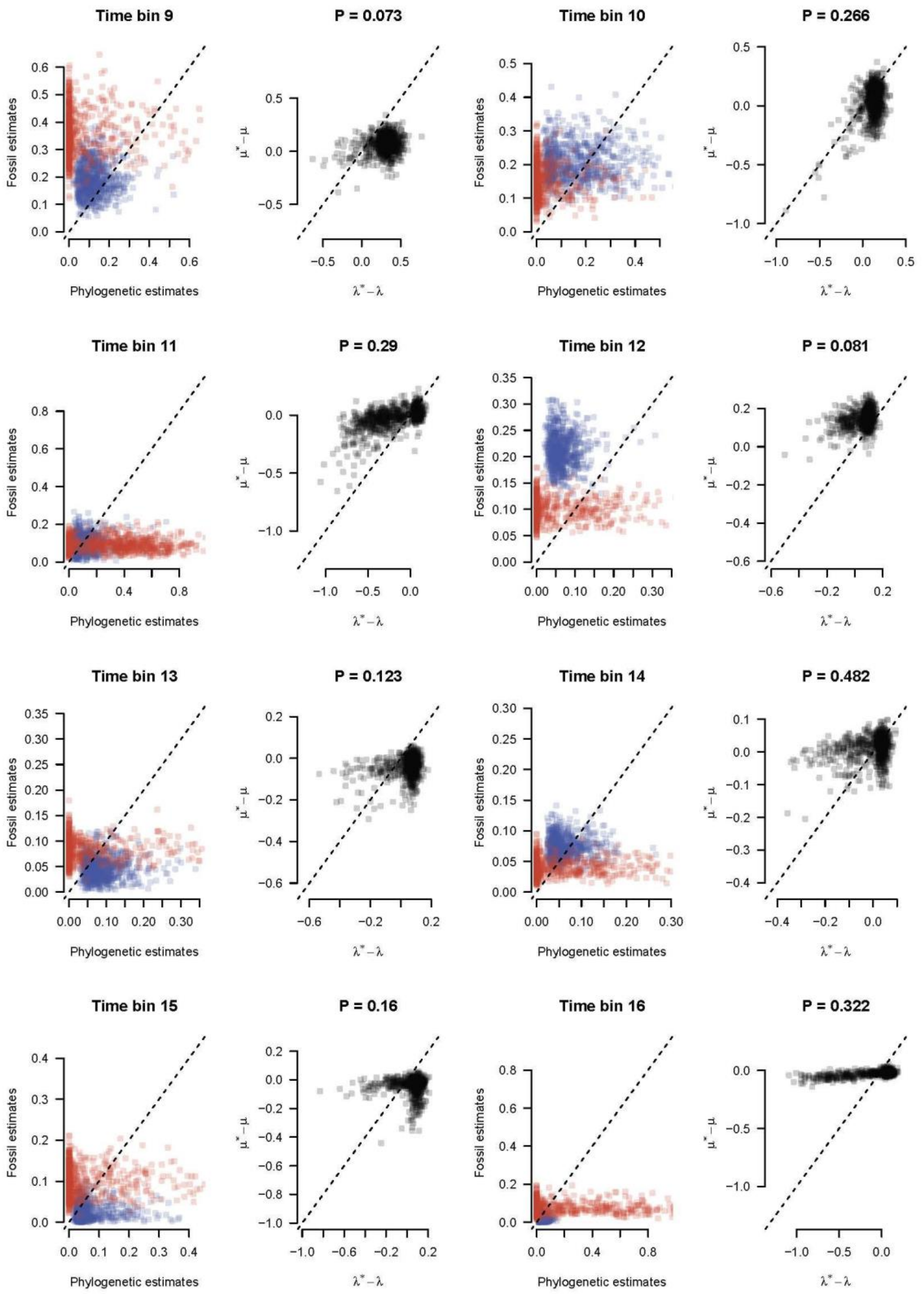

Figure S2. Results from a joint Bayesian analysis of fossil and phylogenetic data for carcharhiniforms under the BDC time-variable model. Posterior samples of speciation rates (in blue) and extinction rates (in red) jointly inferred from the two data types are plotted against one another; posterior samples of the two terms are shown in black. Sixteen rate shifts were used to account for rate heterogeneity. Under this model, the carcharhiniform phylogenetic and fossil data conform to the BDC model, which also conforms to the assumption of constant rates (Fig. S1).

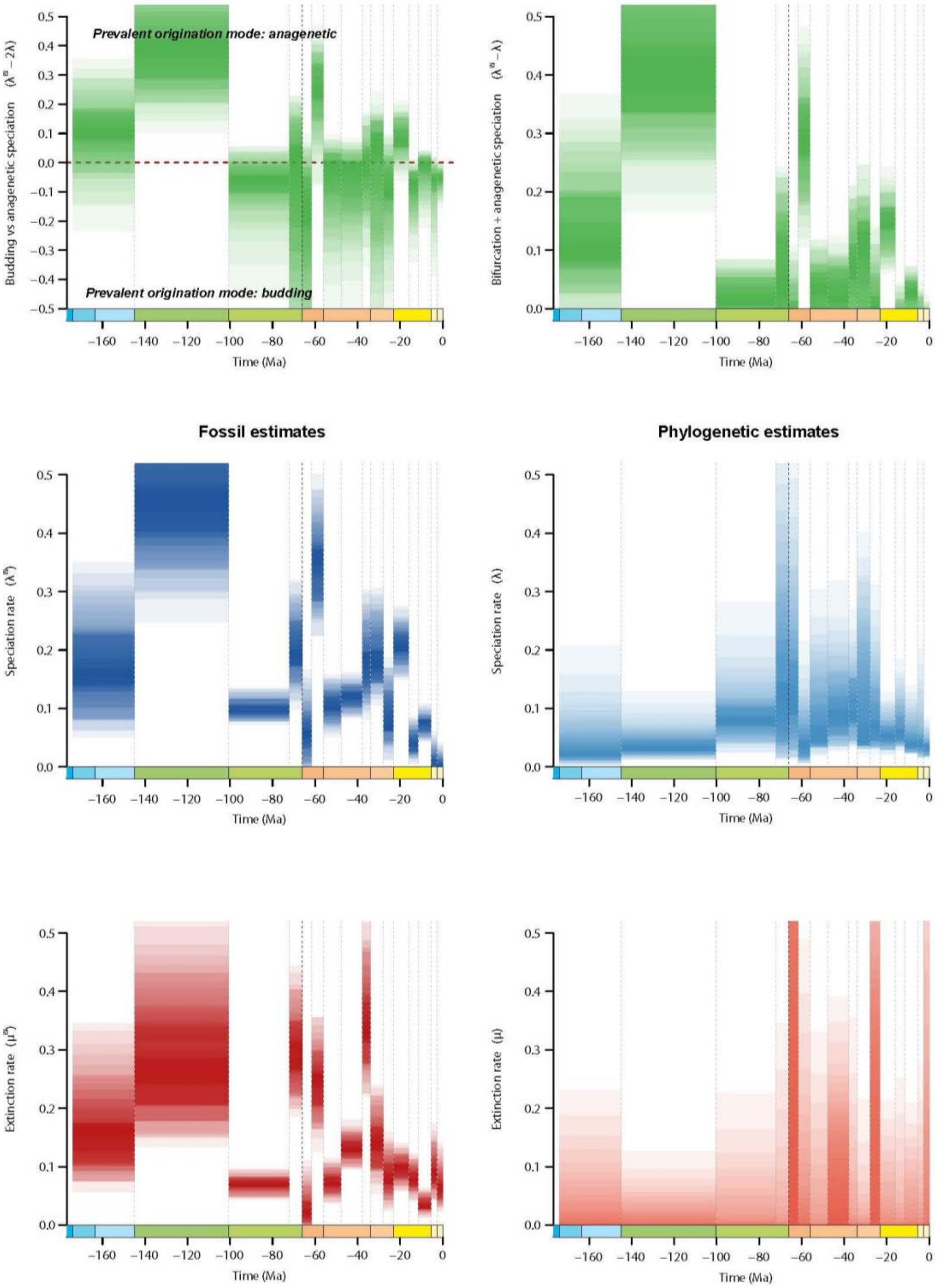

Figure S3. Analysis of the carcharhiniform fossil dataset under the BDC time-variable model. Speciation and extinction rates were jointly inferred within each time bin from the fossil record and phylogenetic tree, under the constraints imposed by the BDC model. Prevalent modes of origination (in green) indicate that, as expected for a species-level data set, budding was more important than anagenetic speciation, and that there is a low but non-zero rate of bifurcation and anagenesis. Speciation and extinction rates inferred from fossils and from the phylogeny of carcharhiniforms show a substantial amount of variation through time with a general tendency to decrease over time, although the net diversification remains positive in most of the time bins except around the Cretaceous/Paleogene and Eocene/Oligocene boundaries when extinction exceeded speciation.

Reference

Silvestro, D., Warnock, R., Gavryushkina, A., & Stadler, T. (2018). Closing the gap between palaeontological and neontological speciation and extinction rate estimates. *Nature Communications*, 9, 5237.
