# Supplementary material for: Combining palaeontological and neontological data shows a delayed diversification burst of carcharhiniform sharks likely mediated by environmental change
Source: Sci Rep. 2022 Dec 19;12:21906. doi: 10.1038/s41598-022-26010-7 (PMC9763247; doi:10.1038/s41598-022-26010-7)
Supplement: Supplementary file 21 — Supplementary Information 21. [file 41598_2022_26010_MOESM21_ESM.pdf]

Supplementary Data S21. Estimating palaeodiversity with mcmcDivE in PyRate.

Method

Statistical methods for estimating palaeodiversity cannot consider uneven sampling due to variations in preservation, biased collection efforts and description rates, which are highly dependent on the study clade. Flannery-Sutherland et al. (2022) presented the mcmcDivE method to infer "corrected" palaeodiversity curves based on the fossil occurrences and on the preservation rates (*q*) through time and across lineages as they can be inferred with PyRate (BDCS or RJMCMC models). The mcmcDivE method implements a hierarchical Bayesian model to estimate corrected diversity across arbitrarily user-defined time bins. As a result, the mcmcDivE method estimates two parameters: (1) the number of unobserved species for each time bin, and (2) a parameter quantifying the volatility of the diversity trajectory (i.e. the degree of variation of diversity). To do so, mcmcDivE assumes that the sampled number of taxa in a time bin is a random subset of an unknown total taxon set. The mcmcDivE methods thus aims at estimating the "true" palaeodiversity curve. Flannery-Sutherland et al. (2022) showed that the relative changes in diversity-through time can be accurately estimated, despite the absolute estimates of diversity are on average less accurate in the presence of strong rate heterogeneity across lineages (in addition to strong rate variation through time).

To compare the palaeodiversity dynamics estimated with the *BDCS-Fossils* and *BDCS-Combined* (Fig. 5), we inferred the carcharhiniform species-level palaeodiversity with mcmcDivE as implemented in PyRate 3 (Flannery-Sutherland et al. 2022). As input, mcmcDivE requires the species-level fossil occurrence data (-*d* option), the time bins used to infer preservation rates (-*q* option), and the posterior samples of the preservation rates inferred from previous PyRate analyses (-*m* option). With the BDCS model in PyRate, we analysed the species-level fossil occurrence data (see *Methods* in the main text) to estimate both preservation rates through time and the amount of rate heterogeneity across lineages using the TPP preservation rate model (-*qShift* option) with gamma-distributed heterogeneity (-*mG* option). The mcmc.log files are used as input for the -*m* option in mcmcDivE. We ran mcmcDivE for 20,000,000 MCMC iterations stating the current standing diversity of Carcharhiniformes (-*N 284* option) and assuming bins of 3-Myr duration (-*b 50* option) to estimate corrected diversity trajectories while resampling the posterior distributions of the preservation parameters inferred by the BDCS model of PyRate (from the mcmc.log files). We used the R function *plot_mcmcDivE_results* to plot and summarize the estimated palaeodiversity trajectories by calculating the median of the posterior samples and the 95% HPD per bin.

Results

The mcmcDivE analyses converged very well (effective sample size >> 200 for all parameters). The parameter quantifying the volatility of the diversity trajectory (*sig2_hp*) is estimated to be low (median = 0.065, 95% HPD = 0.0318, 0.1225), meaning that palaeodiversity dynamics is accurate. Based on the mcmcDivE inferences (central plot on Fig. S1 below), the palaeodiversity dynamics (number of species for each time bin) match well with the palaeodiversity dynamics as inferred with that from the *BDCS-Fossils* (left plot on Fig. S1 below). Nonetheless, we observe higher diversity peaks with the mcmcDivE method in the Late Cretaceous and middle Eocene, suggesting that there are likely unobserved taxa (not yet sampled). However, and more importantly, the mcmcDivE palaeodiversity dynamics do not recover the major radiation of Carcharhiniformes starting in the Neogene captured with the *BDCS-Combined* (right plot on Fig. S1 below). Interestingly, the *BDCS-Combined* compensates the diversity gaps (unobserved taxa) of the Eocene compared to the *BDCS-Fossils*, which makes the *BDCS-Combined* more in line with the mcmcDivE method.

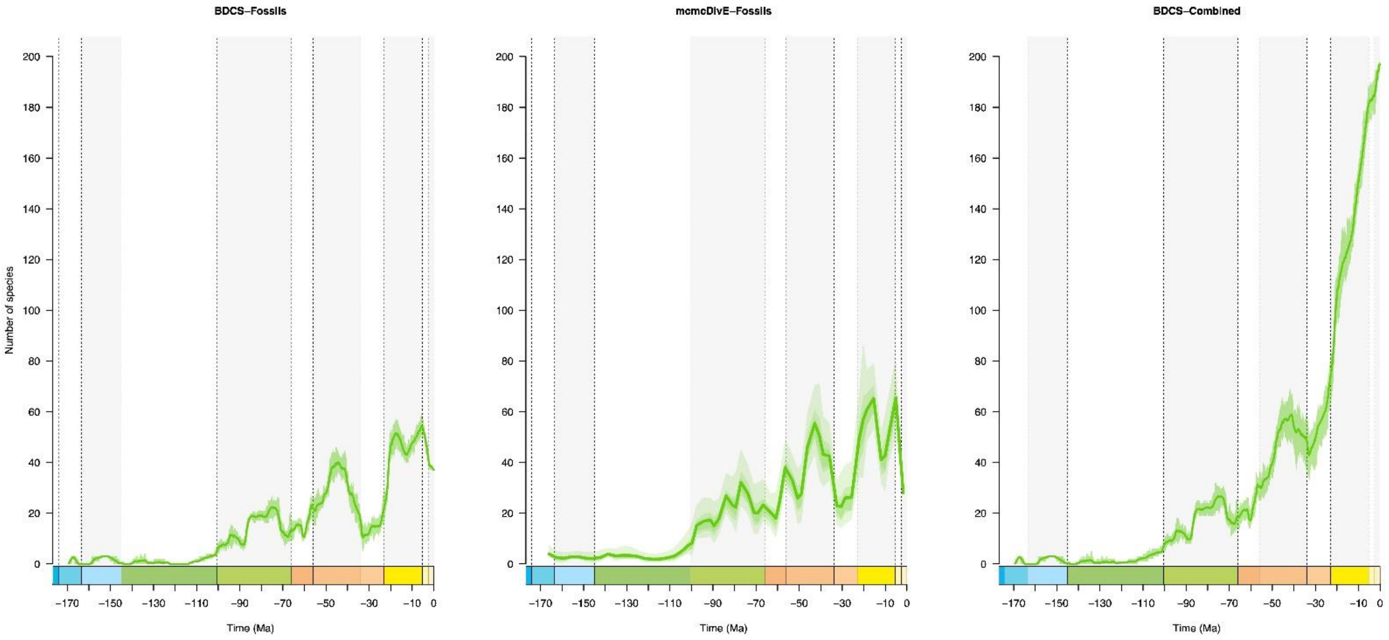

Figure S1. Comparison of the palaeodiversity dynamics as inferred with the BDCS-Fossils (without correcting for non-sampled taxa), the mcmcDivE (correcting for non-sampled taxa), and the BDCS-Combined (not correcting for non-sampled taxa but including extant species from the molecular phylogeny).

Reference

Flannery-Sutherland, J. T., Silvestro, D., & Benton, M. J. (2022). Global diversity dynamics in the fossil record are regionally heterogeneous. *Nature Communications*, 13, 2751.
